# Comprehensive machine-learning-based analysis of microRNA–target interactions reveals variable transferability of interaction rules across species

**DOI:** 10.1186/s12859-021-04164-x

**Published:** 2021-05-24

**Authors:** Gilad Ben Or, Isana Veksler-Lublinsky

**Affiliations:** Department of Software and Information Systems Engineering, Ben-Gurion University of the Negev, Beer Sheva, Israel

**Keywords:** Machine learning, miRNA, Target prediction, CLASH, AGO-CLIP, Chimeric miRNA–target interactions, Cross-species prediction

## Abstract

**Background:**

MicroRNAs (miRNAs) are small non-coding RNAs that regulate gene expression post-transcriptionally via base-pairing with complementary sequences on messenger RNAs (mRNAs). Due to the technical challenges involved in the application of high-throughput experimental methods, datasets of direct bona fide miRNA targets exist only for a few model organisms. Machine learning (ML)-based target prediction models were successfully trained and tested on some of these datasets. There is a need to further apply the trained models to organisms in which experimental training data are unavailable. However, it is largely unknown how the features of miRNA–target interactions evolve and whether some features have remained fixed during evolution, raising questions regarding the general, cross-species applicability of currently available ML methods.

**Results:**

We examined the evolution of miRNA–target interaction rules and used data science and ML approaches to investigate whether these rules are transferable between species. We analyzed eight datasets of direct miRNA–target interactions in four species (human, mouse, worm, cattle). Using ML classifiers, we achieved high accuracy for intra-dataset classification and found that the most influential features of all datasets overlap significantly. To explore the relationships between datasets, we measured the divergence of their miRNA seed sequences and evaluated the performance of cross-dataset classification. We found that both measures coincide with the evolutionary distance between the compared species.

**Conclusions:**

The transferability of miRNA–targeting rules between species depends on several factors, the most associated factors being the composition of seed families and evolutionary distance. Furthermore, our feature-importance results suggest that some miRNA–target features have evolved while others remained fixed during the evolution of the species. Our findings lay the foundation for the future development of target prediction tools that could be applied to “non-model” organisms for which minimal experimental data are available.

**Availability and implementation:**

The code is freely available at https://github.com/gbenor/TPVOD.

**Supplementary Information:**

The online version supplementary material available at 10.1186/s12859-021-04164-x.

## Background

MicroRNAs (miRNAs) are small non-coding RNAs that regulate gene expression post-transcriptionally. miRNAs are encoded in the genome and are generated in a multi-stage process by endogenous protein factors [1]. The mature, functional miRNAs associate with Argonaute proteins to form the core of the miRNA-induced silencing complex (miRISC). miRISC uses the sequence information in the miRNA as a guide to recognize and bind partially complementary sequences on the 3’ untranslated region (UTR) of target mRNAs. miRISC binding typically leads to the translational inhibition and/or the degradation of targeted mRNAs [2]. miRNAs are evolutionarily conserved and are present in the genomes of animals, plants and viruses [3]. miRNAs have diverse developmental and physiological functions and they have been implicated in numerous human diseases [4].

The identification of miRNA target sites on mRNAs is a fundamental step in understanding the involvement of miRNAs in cellular processes. Several experimental high-throughput methods for identifying miRNA targets have been developed in recent years [5, 6], of which the most common and straightforward approach is based on measuring changes in mRNA levels following miRNA over-expression or inhibition in tissue-cultured cells [7]. However, this approach has several major limitations [5, 6]. First, such data may contain indirect signals of miRNA regulation from the downstream genes of direct miRNA targets. Second, for direct regulation, the exact sequences of binding sites are unknown and must be predicted within the relevant mRNA sequence. Third, such experimental settings may represent a non-physiological context for miRNA activity, which does not reflect endogenous targeting rules. Finally, this approach may miss signals of translation-efficiency inhibitions, which affect gene expression but are not reflected in changes in mRNA levels [8].

Other methods, e.g., HITS-CLIP [9, 10] and PAR-CLIP [11], are based on the crosslinking and immunoprecipitation (CLIP) of RNA–protein complexes that are found in direct contact. The crosslinked complexes are immunoprecipitated with a specific AGO antibody (AGO-CLIP) and the associated miRNAs and mRNA targets are collected for further sequencing analysis. While these methods greatly decrease the target search space to precise regions on mRNAs, the identity of the specific miRNA engaged in each interaction is unknown and needs to be predicted bioinformatically [12, 13], e.g., by identifying which highly expressed miRNAs are associated with individual AGO-CLIP peaks [14–17].

Recently, more advanced methods, e.g., cross-linking, ligation and sequencing of hybrids (CLASH) [18], covalent ligation of endogenous Argonaute-bound RNAs (CLEAR)-CLIP [19, 20], and modified iPAR-CLIP [21] have been developed to capture miRNAs bound to their respective targets. These methods are derived from AGO-CLIP and use an extra step to covalently ligate the 3’ end of a miRNA and the 5’ end of the associated target RNA within the miRISC. Subsequent cloning and sequencing of isolated chimeric miRNA–target reads facilitate the identification of direct miRNA–target interactions. Using these methods, datasets of chimeric miRNA–target interactions were generated from cells originating from human, mouse, the nematode *Caenorhabditis elegans*, and the cattle *Bos taurus*. An additional method, iCLIP [22], was applied to *C. elegans* to recover chimeric sequences without employing the ligation step. Furthermore, a re-analysis of published human and mouse AGO-CLIP data revealed additional chimeric miRNA–target interactions in libraries where no ligase was added [21].

The analysis of chimeric miRNA–target interactions from the above-mentioned studies revealed that many of them display non-canonical seed binding patterns and involve nucleotides outside of the seed region. Despite the great contribution that these experimental methods can bring to the field of miRNA, their application is technically challenging. Therefore, to date, datasets have been generated for only a small number of model organisms (Table 1).

The limited number of experimentally identified miRNA–target interactions has promoted the use of computational predictions to expand the miRNA–target repertoires. However, computational identification is very challenging because miRNAs are short and engage only a partial sequence complementarity to their targets, and the rules that govern the miRNA targeting process are not, yet, fully understood. Over the past 15 years, many computational tools have been developed for miRNA target prediction. The first generation of these tools was based on very general rules of thumb, e.g., canonical seed pairing, miRNA–target duplex energy, conservation of the target site, and accessibility (e.g., RNAhybrid [23], miRanda [24], TargetScan [25], and PITA [26]). These tools suffer from high false positive and false negative prediction rates [27–30] due to the limitations of general rules and insufficient knowledge about seedless interactions and base-pairing patterns in the non-seed region. In addition, the target prediction outputs of various tools only partially overlap, hindering the choice of candidates for further experimental validation or more global downstream analysis.

The accumulation of experimentally validated miRNA targets in public databases such as miRecords [31] and miRTarBase [32] has led to the development of new machine-learning (ML) based methods for miRNA target prediction, e.g., SVMicrO [33], SMILE [34], mirMark [35], MiRTDL [36], and deepTarget [37]. Recently, a method that is based on a recommendation algorithm that focuses on network-based inference, miRTRS [38], was developed for miRNA–target prediction.

The availability of new datasets of high-throughput, direct miRNA–target interactions (e.g., [18–21]) has led to the additional development of ML-based methods that incorporate, in their training phase, chimeric miRNA–target interactions [39–45]. These ML-based methods are designed to capture both canonical sites (based on seed complementarity) and non-canonical sites with pairing beyond the seed region. They incorporated tens to hundreds of different features in their models to represent e.g., the sequence, structure, conservation, and context of the interacting molecules, and they were reported to achieve significant improvement in overall predictive performance, as compared with earlier tools. Differences in several aspects can be observed among ML-based methods, including the ML approach and the features used, the choice of datasets for training and testing, the inclusion or exclusion of non-canonical interactions from the training/testing set, and the approach of generating negative data. We provide a summary of some of the above-mentioned methods, focusing on these aspects, in the supplementary material (Additional file 1: Section 1 and Table S1). Briefly, the methods chimiRic [39] and miRTarget [44, 45] use support vector machine (SVM) to classify miRNA–target interactions; TarPmiR [40] is a random-forest- (RF) based approach that provides the probability that a candidate target site is a true target site; and DeepMirTar [42], miRAW [41], and mirLSTM [43] apply deep-learning approaches that are based on stacked de-noising auto-encoder (SdA), deep artificial neural networks (ANN), and long short term memory (LSTM), respectively.

In these methods, the ML models were trained and tested on a dataset of chimeric interactions from human cells generated with the CLASH method [18]. In some of the studies, the dataset was filtered based on the location of the sites, seed pairing pattern, or functional evidence; in others, it was complemented with additional interactions from other experiments. For example, DeepMirTar [42] and mirLSTM [43] included only canonical and non-canonical sites that are located at the 3’UTRs and added interactions that were retrieved from miRecords [31]. chimiRic [39] and miRAW [41] complemented this dataset with seed-containing sites from AGO-CLIP data, while miRTarget [44] complemented the dataset with endogenously ligated chimeras from human AGO-CLIP experiments. miRAW [41] and miRTarget *v4* [45] intersected the CLASH dataset with other resources to retain only the interactions with functional evidence.

For additional independent testing, the above-mentioned methods used few other datasets that are not necessarily derived from ligation-based experiments. These datasets include human PAR-CLIP datasets, mouse HITS-CLIP dataset, chimeric interactions from iPAR-CLIP in *C. elegans* and CLEAR-CLIP in mouse [19], and microarray-based datasets following miRNA transfections or knockdowns (Additional file 1: Table S1). Several public databases, such as miRWalk [46], miRNet [47], and miRDB [48], provide predictions for miRNA–target interactions produced by the mentioned ML-based models.

To date, the experimental datasets that are used to train the ML-based tools are limited to only a few model organisms. Nevertheless, there is a need to apply target prediction tools to other species also, for which experimental data is unavailable. Although some of the existing ML methods examined the possibility to predict interactions in species that are different from the species on which they were trained (e.g., [39, 40, 45]), in all cases the training was performed on human datasets and was applied on only a few other species. The ability of ML-based methods to provide predictions in the opposite direction (namely, from non-human species to human), or between species other than human, was not tested. Moreover, it is largely unknown how the patterns of miRNA–target interactions evolve across bilaterian species and whether some features remained fixed throughout the evolution of the species, raising questions regarding the general, cross-species applicability of currently available ML methods.

**Fig. 1:**
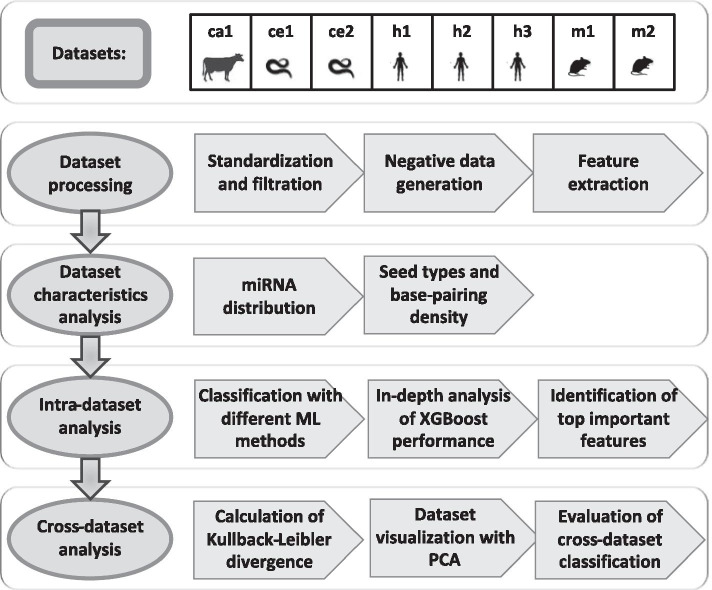
A flowchart depicting the outline of the study. Overall, eight publicly available datasets of chimeric miRNA–target interactions were used in this study, including one from cattle (ca) *B. taurus*, two from the worm *C. elegans* (ce), three from humans (h), and two from mice (m). The study included four main steps. The first three steps (processing, characterization and classification) were applied separately on each dataset; in the fourth step, the relationships between datasets were examined

In this study, we used available datasets of high-throughput direct miRNA–target interactions to determine whether miRNA–target interaction rules are transferable across species. A flowchart describing the steps taken in this study is depicted in Fig. 1. We evaluated eight datasets from four species (human, mouse, worm, and cattle), generated from various tissues and experimental protocols. We developed a processing pipeline to transform these datasets into a standard format that enables their comparison and integration. We provide a detailed overview of the datasets, focusing on their size, miRNA-seed family composition, and interaction patterns, highlighting their resemblance and dissimilarity. For each dataset, we trained and tested six commonly used ML classifiers for the prediction of miRNA–target interactions and evaluated the importance of the features we used. We then explored the relationships between datasets by measuring the divergence of their miRNA seed sequences and by evaluating the performance of cross-dataset classification. Our findings indicate that the transferability of miRNA-targeting rules between different species depends on several factors, including the composition of seed families and the evolutionary distance. Our study provides important insights for the future development of target-prediction tools that could be applied to species for which experimental data is limited.

## Results

### Dataset processing

Eight miRNA–target chimera datasets have been previously generated for human, mouse, worm (*C. elegans*), and cattle (*B. taurus*). The details of each dataset are provided in Table 1, including the species, the cell type or developmental stage that was examined, and the experimental methods used to obtain the data. We applied a multi-step pipeline to process and filter these datasets (see Methods: Data processing). The numbers of interactions that passed the pipeline stages are shown in Table 2. Of all the interactions in the datasets, 3’UTR interactions constitute 10–47%; among them, interactions with either canonical or non-canonical seed-pairing constitute 53–82%. The pipeline produced final datasets of various sizes: four small datasets (500–1200), two large datasets (2000–5000), and two massive datasets ($\sim$18,000 each). As these final datasets were later used as input for machine learning (ML) tasks, we complemented them with synthetically generated negative interactions, as described in the Methods: Generation of negative interactions. We extracted 490 features from each interaction, representing the properties of the interaction duplex and of the interaction site and its flanking region within the 3’UTR (see Methods: Features).

### Dataset characteristics

In the following subsections, we characterize the interactions of each dataset, based on its miRNA distribution and base-pairing patterns. Since the negative interactions are generated synthetically, we focus on positive interactions.

#### miRNA distribution

**Fig. 2:**
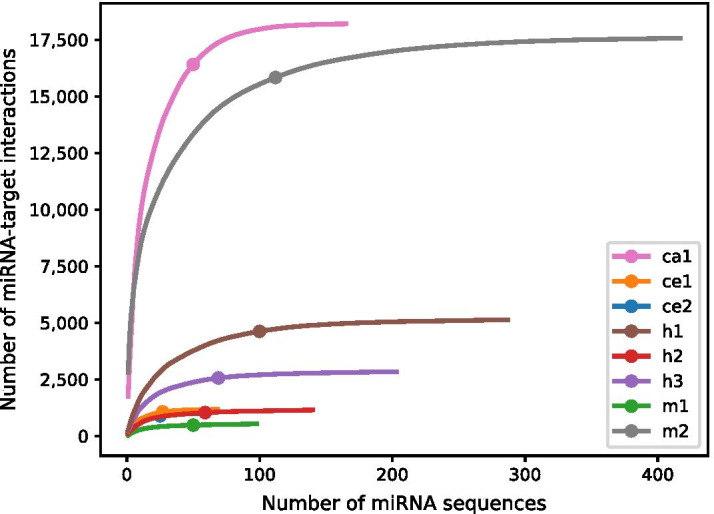
Cumulative sum of miRNA sequence appearances in the examined datasets. Each curve corresponds to the cumulative sum of one of the datasets, where the minimum number of unique miRNA sequences needed to represent 90% of the interactions within the dataset is indicated by a filled circle. The height of each curve represents the size of the dataset and its width represents the number of unique miRNA sequences that comprise it

We counted the appearance of miRNA sequences and miRNA seed families (nt2-7) and generated a distribution function for each dataset (Table 3). Our analysis indicates that the datasets are not uniformly distributed in terms of miRNA appearances (Fig. 2). Furthermore, 90% of the interactions are dominated by a small subset of miRNA sequences (25–50%) or miRNA seed families (18–37%).

#### Seed types and base-pairing density

**Fig. 3:**
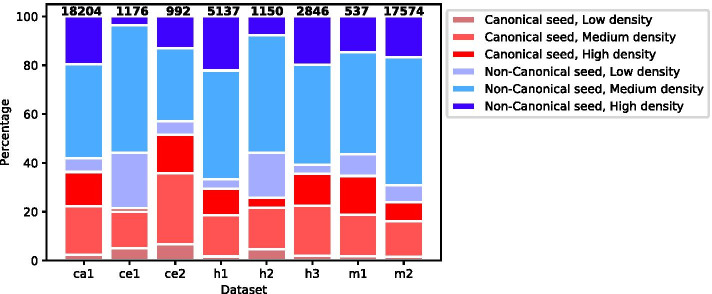
Classification of the miRNA–target duplexes, based on their base-pairing patterns. Distribution of miRNA–target duplexes across six classes according to the seed type (canonical or non-canonical) and the base-pairing density (low: < 11 bp, medium: 11–16 bp, or high: > 16 bp). The number above each bar indicates the total number of interactions in the dataset

We classified the interactions (i.e., the corresponding duplexes formed by the miRNA and the target site) based on two parameters: seed type (canonical or non-canonical, see Methods) and base-pairing density [number of base-pairs (bp) within the duplex: low < 11 bp, medium: 11–16 bp, and high:> 16 bp]. We defined six classes, based on combinations of seed type and base-pairing density, and assigned each interaction to the appropriate class (Fig. 3). As can be seen in the figure, the datasets are rich, diverse, and include all the combinations of seed type and base-pairing density. However, two observations stand out: first, in terms of seed type, most interactions (48–70%) are non-canonical; and second, for both seed type classes, most interactions demonstrate either medium or high base-pairing density, while the low base-pairing density interactions comprise only a small portion of the datasets. A similar analysis for the negative interactions is shown in Additional file 1: Figure S1.

### Intra-dataset analysis

In this section, we evaluate the performance of ML-based binary classifiers to correctly classify positive and negative miRNA–target interactions within the same dataset. We first conducted a set of experiments with different types of commonly used ML classifiers, and then we performed an in-depth analysis of the best-performing classifier by measuring different performance metrics and by estimating feature importance.

#### Evaluation of different machine-learning methods

For each dataset, we generated 20 training-testing splits of the data, using a stratified random-split algorithm. This split algorithm ensures that each miRNA appears in both the training and the testing sets in the same proportion as in the original dataset. We then trained six widely used classifiers on the 20 training sets of each dataset and measured their performance in the classification of their respective testing sets. We calculated the means and standard deviations of the classification accuracy, as shown in Table 4. Notably, the XGBoost classifier achieved the best results across all datasets, with accuracy scores ranging from 0.82 to 0.94, with the following order of performance from low to high: *h1*, *h3*, *m1*, *ce1*, *ce2*, *m2*, *h2*, and *ca1*. We did not observe any bias in the ordering of the species in the list. We compared our results to previous ML-based approaches that were trained and tested on the human CLASH dataset designated as *h1* in Table 1. The accuracy achieved by our classifiers for this dataset is comparable to those reported in previous studies (Additional file 1: Table S2).

#### In-depth analysis of the XGBoost performance

As XGBoost achieved the highest classification accuracy across all datasets, we next conducted an in-depth performance analysis for this classifier, calculating five additional commonly used performance metrics: sensitivity (true positive rate, TPR), specificity (true negative rate, TNR), area under the curve (AUC), the Matthews correlation coefficient (MCC), and the F1 score.

We found that the performance metrics of the XGBoost classifiers were similar and relatively high for all datasets (Table 5). As described above, we calculated the means and standard deviations of all metrics across 20 training–testing data splits. The average scores ranged as follows: AUC: 0.91–0.98, TPR and TNR: 0.82–0.91, MCC: 0.65–0.87, and F1 score: 0.82–0.94. In accordance with the accuracy metric calculated above, these findings indicate that all eight XGBoost classifiers (corresponding to each dataset) are accurate, balanced, and precise.

#### Top important features of each dataset

**Fig. 4:**
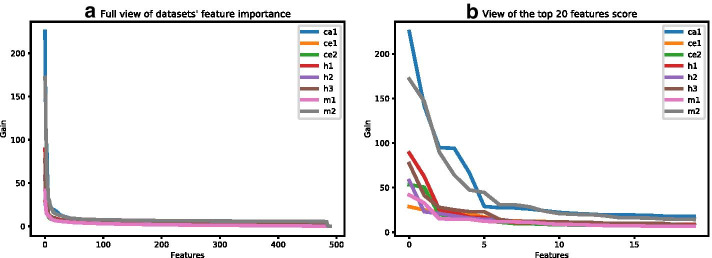
Dataset feature importance plot based on gain score. The features are sorted in descending order of importance, from the highest importance (highest gain) to the lowest. **a** A full view of the gain plot, emphasizing the gain decay. **b** A zoomed-in view, focusing on the 20 most important features

Next, of the 490 features that we used to describe the interactions, we sought to identify the top important features of each dataset, their relative scores, and the degree of overlap of the top features between different datasets. The XGBoost classifier provides a list of five feature-importance metrics: weight, gain, cover, total gain, and total cover. We extracted these five metrics for all 20 training–testing splits of each dataset and calculated their means and standard deviations (Additional file 2: Table S8). Of these metrics, we chose the gain metric, which reflects the contribution of each feature to the model, for further analysis. For each dataset, we sorted the features in descending order, based on their mean gain score. The plots of the feature-importance curves for all datasets are shown in Fig. 4. These analyses indicate that the gain score decays very fast (Fig. 4a) and that the top six features are significantly stronger than the other features (Fig. 4b). Therefore, we extracted the top six features from each dataset, along with their scaled gain score (see Methods), into a unified list. This unified list consisted of 16 features in total (out of the maximum length of 48 features), indicating that many features are shared among the datasets. Table 6 shows the features ordered by their mean gain across all datasets, and the top six features of each dataset are marked with a star. At least 3 features are common to each dataset pair, and only a small number of features belong to a single dataset, indicating that the features in the unified list may well represent all eight datasets. Notably, features related to the seed region (marked with italic font in the table) comprise half of the features in the list. This finding emphasizes the role of the seed region in the formation of miRNA–mRNA interactions.

### Cross-dataset analysis

In the previous section, we trained, optimized, and evaluated the performance of a dedicated classifier for each dataset. Here, we examine the relationships between datasets. To that end, we first used a statistical measure to calculate the distance between the datasets and then visualized the datasets based on the unified list of the 16 most important features that were reported above (Table 6). Finally, we evaluated the performance of each dataset-specific classifier in properly classifying the interactions in other datasets.

#### Kullback–Leibler divergence

We hypothesized that a classifier will perform better when it is tested on a dataset whose characteristics are similar to those of the training dataset than on a dataset with different characteristics. We thus looked for a measure to assess the level of similarity between pairs of datasets, which will consider the directionality of the classification task: the classifier is trained on one dataset (the source) and is applied to classify a second dataset (the target). We chose to use Kullback–Leibler (KL) divergence: an asymmetric measure of the difference between the probability distributions of the target and the source datasets. KL divergence, whose origins are in information theory, is widely used to measure information loss and thereby assess the degree to which samples from one distribution can be approximated by samples from another distribution (e.g., in cases where a simple distribution, such as a uniform or a Gaussian distribution, approximates experimental data).

Here, we used the KL divergence to measure the pairwise information loss between each of the two datasets that will be used, in the analysis described below, as the training and testing sets. For each pair, the calculated KL divergence can be interpreted as the amount of information lost when the training set represents the testing set. The divergence is calculated based on the miRNA seed family distribution of the datasets (see Methods: "Calculation of the Kullback–Leibler divergence").

**Fig. 5:**
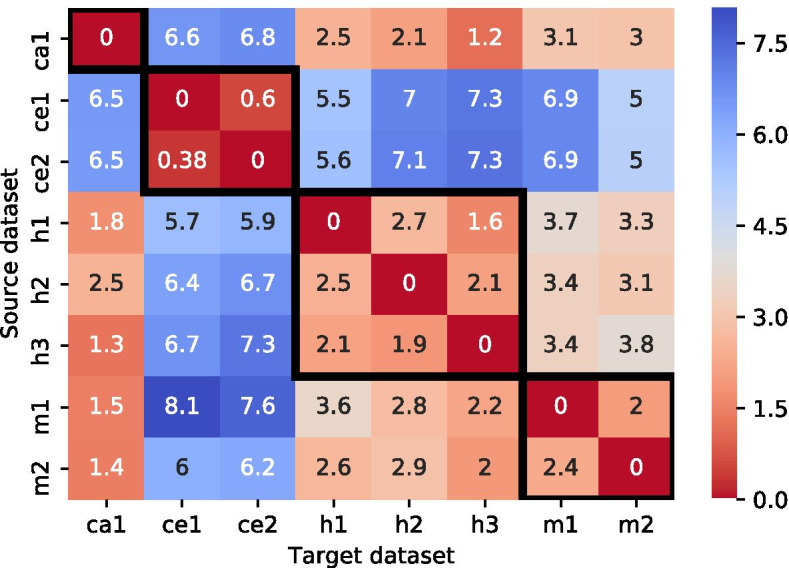
Kullback–Leibler (KL) divergence of all dataset pairs. Each cell *(i,j)* represents the divergence from a source dataset *i* to a target dataset *j* (KL(j || i)), based on their miRNA seed family distributions. The black frames indicate the results of dataset pairs originating from the same species

Figure 5 shows the divergence between each pair of datasets. The divergence of a dataset with itself is zero and the divergence between datasets within the same species is typically lower than the divergence between different species. Notably, the divergence between the *C. elegans* datasets–either as targets or as sources–and the other datasets is significantly higher (range 5–8.1) than the divergence between other pairs (range 1.2–3.8), indicating that seed distributions of other species poorly represent the *C. elegans* datasets, and vice versa. The asymmetry of the KL divergence can be observed, for example, in the pair *(h1,h3)*, for which *KL(target = h3 || source = h1) = 1.6* and *KL(h1 || h3) = 2.1*. Intuitively, this finding means that dataset *h1* better approximates dataset *h3* and that information loss is smaller than in the opposite case.

#### Dataset visualization

**Fig. 6:**
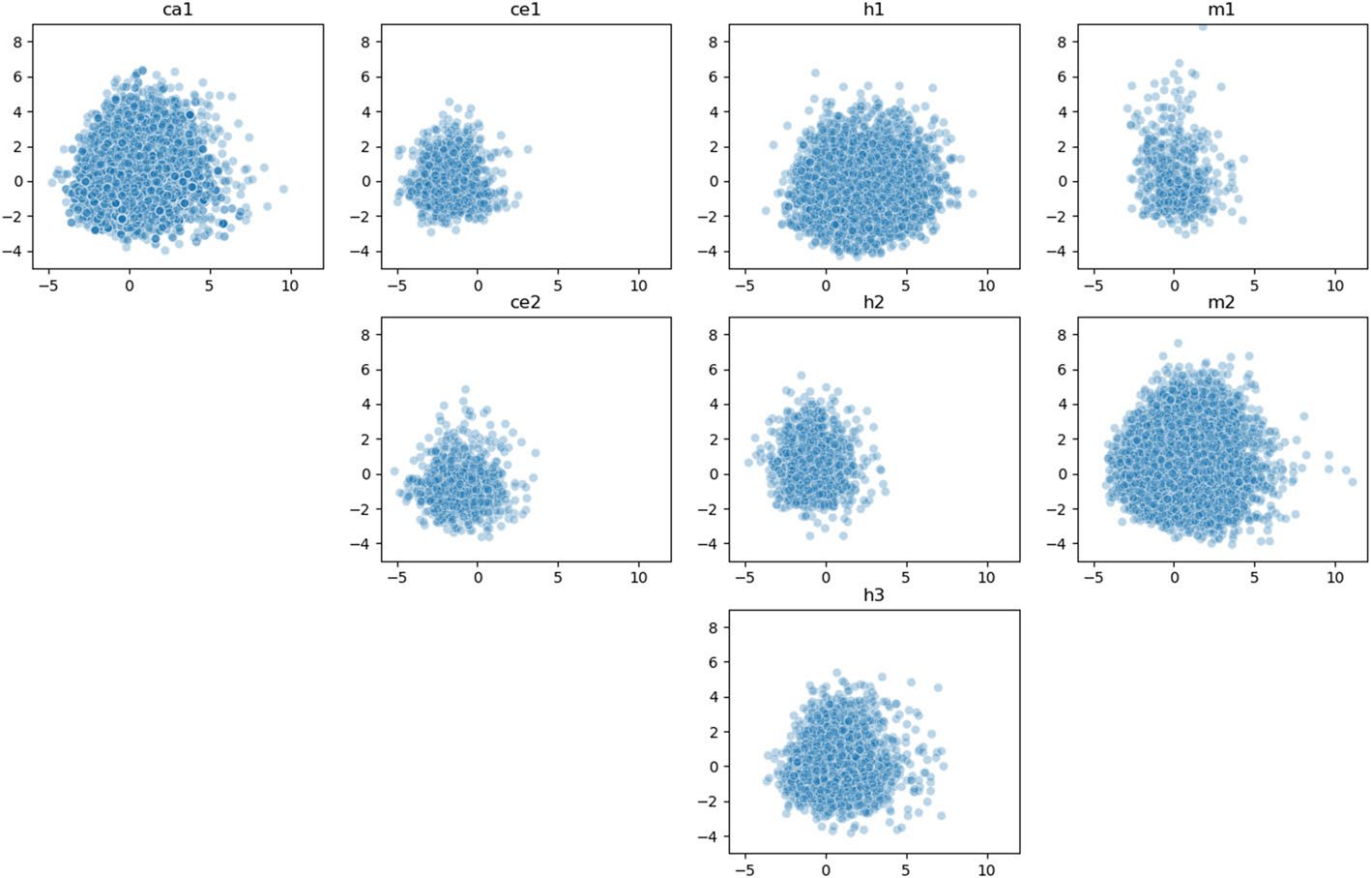
Two-dimensional visualization of the datasets. Each point represents a single positive interaction after a dimensional reduction of its features’ space using PCA. The X and Y axes are the first and the second components of the PCA, respectively

Visualization is an important step in the analysis of high-throughput biological data and can assist in revealing hidden phenomena. However, visualization is challenging when the data are represented by a large number of features. A dimensionality reduction algorithm enables the representation of the data in a 2-dimensional scatter-plot and facilitates the visual inspection of the data. To visualize the datasets in two dimensions, we focused on the experimental interactions of each dataset (the positive data). For each interaction, we selected the top 16 features from the unified list described above (Table 6) and performed a dimensionality reduction using the principal component analysis (PCA) technique. The results are shown in Fig. 6.

Figure 6 reiterates the fact that there are big differences in the sizes of the datasets, reflected in the density of the graphs. For example, the size of the human dataset *h1* is more than twice the size of the datasets *h2* and *h3*, and, indeed, its graph is denser. In addition, notable differences can be observed in the 2-dimensional space spanned by each dataset: while the datasets *ca1*, *h1*, *h3*, and *m2* are spread throughout the entire area, the *C. elegans* datasets (*ce1*, *ce2*) and the datasets composed of endogenously ligated chimeras from a mixture of experiments (*h2*, *m1*) are concentrated in a narrower part of the area.

#### Classification performance differences between datasets

**Fig. 7:**
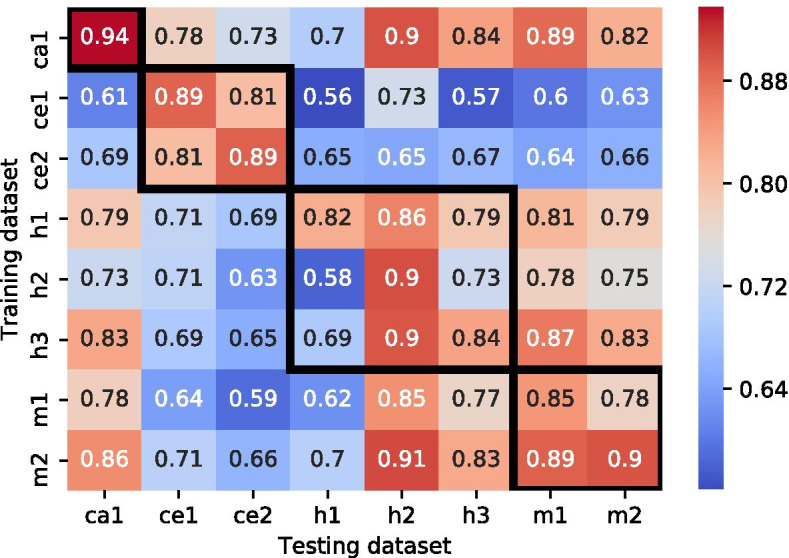
Cross-dataset classification results. Each cell *(i,j)* represents the mean accuracy of the 20 XGBoost classifiers that were trained on dataset *i* (in "Evaluation of different machine-learning methods" section) and tested on dataset *j* (ACC(i, j)). The black frames indicate the results of dataset pairs originating from the same species. The accuracy results for pairs *(i,i)* were taken from "Intra-dataset analysis" section. Note that, for the ease of the interpretation of the results, the color scale is inverse to the scale used for the KL-divergence plot in Fig. 5

We evaluated the performance of cross- dataset miRNA–target predictions, i.e., the performance of a classifier when applied to interactions from datasets different from the one it was trained on. We examined all 56 possible combinations, considering each dataset both as a training set and as a testing set. For each dataset, we loaded the 20 XGBoost classifiers that we trained as described in "Intra-dataset analysis" section and used them to classify the seven remaining datasets. Figure 7 shows, for each pair of datasets, the mean classification accuracy over the 20 tests; the standard deviation values and the results of other ML methods can be found in Additional file 1: Table S6, and Figures S3–S7.

Inspection of the results (excluding the diagonal) reveals variability in the classification performance between the pairs, ranging from random, slightly above 0.5, to 0.91. The accuracy matrix is asymmetric, i.e., a pair in which a dataset *i* serves as a training set and a dataset *j* serves as a testing set achieves a different performance than a swapped pair. Pairs of datasets originating from the same species (indicated by black boxes in Fig. 7) generally achieved higher accuracy than pairs from different species. Intriguingly, the human pairs *(h2,h1)*, *(h2,h3), and* *(h3,h1)* achieved a relatively low accuracy score, which can potentially be explained by the differences in the diversity of the datasets. In particular, the dataset *h2* is smaller and less diverse than datasets *h1* and *h3* (Fig. 6), and thus a model that uses *h2* as the training set achieves lower performance. In most cases, the KL divergence results coincide with the accuracy results. For example, for the pair *(h1,h3)*, the ${KL(h3 || h1) = 1.6 < KL(h1 || h3) = 2.1}$, while ${ACC(train = h1, test = h3) = 0.79 > ACC(h3,h1) = 0.69}$, demonstrating that the dataset *h1* better represents the dataset *h3* and, as such, achieved better accuracy results than vice versa. Notably, the ${KL(h2 || h1) = 2.7 \approx KL(h1 || h2) = 2.5}$, but the ${ACC(h1,h2) = 0.86 > ACC(h2,h1) = 0.58}$. This finding indicates that additional factors—e.g., the patterns of interactions—affect the ability to accurately classify miRNA–target interactions.

Pairs of datasets originating from different species and that included *C. elegans* as either the training or the testing set achieved poor performance, ranging from 0.56 to 0.78. As described above, the divergence scores of these pairs are between two and four times higher (ranging from 5 to 8.1) than the scores of the other pairs. This finding may indicate that the seed distributions of human, mouse, and cattle datasets are not well represented by the seed distributions of the *C. elegans* datasets, and vice versa. Other pairs of two species achieve a much higher accuracy, up to 0.91. The lowest accuracy in these mixed pairs was observed for pairs that contained *h1* as the testing set. Notably, this dataset was used by previously developed methods (reviewed in Additional file 1: Table S1) for training/testing purposes only, and it has never been evaluated as an independent testing set. Additional factors that could influence the classification accuracy are further discussed below.

## Discussion

While the identification of bona fide miRNA targets is crucial for elucidating the functional roles of miRNAs, it remains a major challenge in the field. Novel experimental protocols, which can produce high-throughput, unambiguous interacting miRNA–target datasets, have indeed pushed the field forward in recent years; however, due to technical challenges involved in the application of these methods, there is a constantly increasing interest in using computational approaches for miRNA target prediction, and especially for approaches that are based on advanced ML models. Several studies successfully trained and applied classic ML [39, 40, 44, 45] and deep-learning [41–43] methods to some of the experimental miRNA–target datasets from a few model organisms. However, our limited understating of the evolution of miRNA–target interactions raises questions regarding the applicability of these tools to species for which experimental training data is unavailable.

The ultimate goals of this study were to evaluate the transferability of miRNA–target rules between the examined species and to identify and compare their most influential interaction features. To this end, we systematically characterized the available miRNA–target chimeric datasets and conducted intra- and cross- dataset classification analyses using ML approaches.

### Available data

The availability of large and high-quality datasets is crucial for ML-based research. In the field of experimental miRNA–target identification, several approaches are available for generating high-throughput datasets, each with its own advantages and limitations [5, 6]. In our analysis, we focused on chimeric miRNA–target datasets generated by experimental or endogenous ligation (by, e.g., CLASH [18] or PAR-CLIP [21]), as these datasets provide direct evidence for interactions between a miRNA and a specific target site. Furthermore, these datasets contain many non-canonical interactions, which enrich the repertoire of miRNA–target interactions. On the other hand, the main limitation of ligation-based methods is the low yield of chimeric reads that are recovered ($\sim 2$%), suggesting that many miRNA–target interactions remain uncaptured. In this work, we assume that the captured interactions represent an unbiased sampling of all the interactions in the examined cells. Additional advances in the efficiency of ligation-based methods and deeper sequencing will provide richer datasets, which could be easily incorporated into our analysis for further research.

We utilized eight available chimeric datasets, from four species, which were generated by different experimental protocols. We developed a processing pipeline to transform and unify the different data formats that we encountered during the collection of the datasets. This pipeline is a powerful infrastructure that will enable us, with relatively low effort, to add more data sources to the analysis in the future, when these become available.

### A thorough analysis of the datasets

We characterized the datasets based on their miRNA content and base-pairing patterns. Our analysis of the frequencies of miRNA sequences revealed that there are differences in miRNA sequence distributions between datasets, even if they originated from the same species. In addition, each dataset is dominated by a small set of miRNAs (30–50% of the most frequent miRNAs comprise 90% of all interactions). These distributions mirror the in vivo distributions, as miRNA frequency in miRNA–target chimeras was reported to correlate with total miRNA abundance [19].

We continued categorizing the interactions based on their seed-pairing type (canonical and non-canonical) and base-pairing density. Perfect seed complementarity (referred to as canonical seed pairing) between target sites and miRNA seed sequences (positions 2–7 or 2–8) has long been recognized as a critical dominant feature that determines miRNA targeting efficiency [25, 49, 50]. Nevertheless, in recent years, several examples of functional miRNA–target interactions without perfect seed pairing have been reported, featuring *GU* pairs, mismatches, and bulges in the seed region (referred to as non-canonical seed pairing). Examples include the well-established *let-7* targeting of *lin-41* in *C. elegans* [51, 52], with one site containing a one-nucleotide bulge in the target and the other site containing a *GU* pair. Moreover, non-canonical miRNA–target sites known as “nucleation bulges”, in which the target sites contain a bulged-out *G* in the seed, were identified for *miR-124* when analyzing AGO HITS-CLIP data from the brain of mice [53]. The functionality of non-canonical sites is still a matter of debate. While studies that generated miRNA–target chimeras provided evidence for the functionality of the recovered non-canonical interactions [18, 21], a recent analysis of non-canonical target sites revealed that, although these sites are bound by the miRNA complex, they do not appear to be broadly involved in the regulation of gene expression [54]. Future work will need to focus on generating miRNA functional high-throughput datasets [55] across species, which could be combined with datasets of chimeric interactions, to provide a more robust starting point for similar types of studies.

We showed that the majority (48–70%) of the interactions in most datasets are non-canonical. Furthermore, in both canonical and non-canonical groups, a large fraction of the interactions is characterized by either a medium or a high density of base-pairing (11–16, and >16 base-pairs, respectively), predicting the existence of additional pairing beyond the seed region. These auxiliary non-seed interactions were suggested to compensate for imperfect seed matches [56, 57]. Moreover, non-seed interactions were also shown to contribute to target specificity among miRNA seed family members (same seed, divergent non-seed sequence), both in the case of canonical and of non-canonical seed pairings [19, 22].

### Features and their significance

In this work, we partially adopted the pipeline from DeepMirTar [42], where the interactions are represented by 750 features. These features include high-level and low-level expert-designed features that represent the interacting duplex, sequence composition, free energy, and site accessibility and conservation. Additional raw-data-level features encode the sequences of the miRNA and the target site. We adopted some of the expert-designed features in our study and used a total of 490 different features to describe the interactions, enabling the model to identify and learn different interaction patterns. However, we did not include, the raw-data-level features so as to avoid potential information-leakage from the training set to the testing set for two main reasons. First, we noticed that the miRNA seed families are not uniformly distributed. Second, in our study, the negative sequences were synthetically generated, such that the seed region does not match any annotated miRNA. Accordingly, including raw-data-level features could have led the ML model to learn to distinguish between real and mock miRNA seeds. Moreover, in such a case, the model may be over-fitted and fail to generalize the rules of interactions. Indeed, and perhaps not surprisingly, we achieved higher classification performance by including the raw-data-level features in our models (Additional file 1: Table S4). Another study [41], which used raw sequence features, addressed this issue by generating a negative dataset based on experimentally verified data, instead of using mock miRNAs. A comparison between different methods for the generation of negative datasets is an interesting direction for future research. In particular, the evaluation of how the combination of these methods and different feature sets affects the performance of miRNA–target prediction classifiers would help to generate standard approaches for future studies.

The feature-importance analysis revealed the existence of a small group of significantly dominant features in all datasets. Although the analysis identified the features for each dataset independently, we found a significant overlap between the groups and that the unified group contains only 16 features. Importantly, half of these features are seed-related, reiterating the significance of this region in miRNA–target interactions [54].

Ideally, in ML, we want the ratio between samples and features to be sufficiently high to result in a robust model and to avoid over-fitting. Some of the datasets in our collection are relatively small, with a low ratio of interactions to features; the ratio is $\sim$4 for *ce1, ce2, h2*, and $\sim$2 for *m1*. A low ratio can produce models with high bias and high variance. In general, a reduction in the number of features, when possible, was shown to be a successful practice [58]. In the current study, some of the features are highly correlated and, therefore, can be combined. Several methods for feature selection and dimensionality reduction may be evaluated in the future. As a preview, we used a basic method for feature selection, based on the XGBoost feature importance data. We used the 16 features taken from Table 6 and repeated the classification analysis (Additional file 1: Table S7, Figure S2). The results were similar to the results obtained when all features were included, indicating that future research that will evaluate different dimensionality reduction methods should be considered to optimize the classification models.

### Training and testing dataset split

The procedure of splitting the data into a training and a testing set has a crucial role in the evaluation of ML models. In the miRNA–target prediction task, there is no pre-defined split to training and testing sets, as is common in other fields, such as in computer vision (e.g., MNIST [59]). Therefore, we used three strategies to reduce the effect of the split on our results: (1) using a stratified training–testing split, which ensures the same distribution of miRNA sequences in both the training and testing sets; (2) generating control sets by using a pure-random split algorithm (Additional file 1: Text and Table S3); and (3) generating several training–testing sets by using different random states for the split approaches (1) and (2), and reporting the means and the standard deviations of the results. Indeed, we obtained similar results and very low standard deviation values with both splitting methods, confirming that the split strategies did not bias our results. It should be noted that, in a cross-dataset evaluation, the testing set is taken as a whole, without any split. Thus, the cross-data result is affected only by the quality of the classifier, without any effect of the splitting procedure.

### Using a tree-based classifier

For our thorough analysis, we used XGboost [60], which is one of the leading gradient boosting tree-based tools for classification [61]. As compared with deep-learning, XGboost is less computationally expensive and usually does not require a GPU for training, and it can work with either small or large datasets. Additionally, XGboost provides the ability to evaluate and explain the classification rules and rank the features by their importance. We show that XGboost achieved the best performance as compared with the statistical ML algorithms (e.g., SVM and LR) in both the intra- and cross-dataset analyses (Table 4, Fig. 7 and Additional file 1: Figures S3–S7). Furthermore, the results of XGboost were comparable to those of deep-learning algorithms that were previously applied on the human dataset *h1* [37, 42].

### Cross-dataset analysis

Most previous works trained and tested their predictive models based on a single chimeric miRNA–target dataset (usually *h1*), sometimes complemented by additional experimental data from databases (e.g., [31, 32]) or AGO-CLIP data [39–43]. These models were then evaluated on portions of the data that were excluded from the training set and, in some cases, on a few independent datasets from either the same or other species (Additional file 1: Table S1). The contribution of our work is in providing the first thorough analysis of all available miRNA–target chimeric datasets, outlining their similarities and dissimilarities. Additionally, we explored the ability to learn classification rules from one dataset and apply them to another dataset, considering all possible combinations of dataset pairs. The accuracy results of cross-dataset classification ranged between 0.56 and 0.91. To explain these results, we examined several factors:

(1) Evolutionary distance: We estimated the distance for each pair of species (i.e., the time since the species diverged from their common ancestor; Table 7). Of the four examined species, the mouse and human are the closest to each other, with cattle equally and relatively close to them, while *C. elegans* is the most distant from all. Indeed, the highest accuracy was obtained when we trained and tested datasets from the same species, while the lowest accuracy was obtained when we trained and tested combinations of the *C. elegans* datasets and the datasets from the other species.

(2) Kullback-Leibler (KL) divergence scores: We measured the divergence for each pair of datasets based on their miRNA seed family distribution. Previous analyses of chimeric datasets showed that individual miRNAs are enriched for specific classes of base-pairing patterns [18, 22], suggesting that they may follow different targeting rules. Therefore, differences in the distributions of miRNA sequences in the training sets may lead to biases in the rules learned by an ML model, which could partially explain the high correlation that we found between KL-divergence and classification performance. Interestingly, and perhaps not surprisingly, the KL divergence results coincide with the evolutionary distance between the species, where the *C.elegans* datasets exhibit the highest distance from the datasets of other species. The divergence within the same species is, on average, lower than the divergence between different species. This divergence probably associates with the differences in miRNA distributions among the different cell types or developmental stages from which the datasets were generated.

(3) Area covered by a 2-dimensional feature space: We visualized the datasets by their features in two dimensions using PCA, which highlighted datasets with a lower spread. In particular, the *C. elegans* datasets are exceptionally concentrated in a narrower area. In addition, the datasets *m1* and *h2*, which represent endogenously ligated chimeras from a mixture of AGO-CLIP experiments, are smaller and less spread than other datasets from the same species. The latter may explain the lower accuracy obtained in cross-datasets experiments that employed these datasets as the training sets.

## Conclusions

The accuracy results obtained in our cross-datasets experiments are relatively high when the species are within a certain evolutionary distance, reflecting the ability of the ML model to generalize interaction rules, learned from a specific dataset, into more universal interaction rules. Taken together our findings suggest that target-prediction models could also be applied to species for which experimental training data is limited or unavailable, as long as they are sufficiently close to the species whose data is used for training.

As more miRNA–mRNA interaction datasets become available, they could be processed with our pipeline and incorporated into the cross-dataset analysis. In the future, the expansion of such analyses to other datasets may also provide insights about the evolution of miRNA-targeting and identify both universal and species-specific features.

We speculate that deep learning models may boost classification performance. Several future research directions that are based on deep learning techniques would be important to follow. The first technique is Transfer Learning, which combines the information from several datasets, and could be used to examine the prediction accuracy in close and in more distant species. The second technique is Multitask Learning (MTL), which jointly learns multiple classification tasks, and can benefit from the observation that all the datasets are represented by the same features. MTL is effective when data are limited and high-dimensional, thereby directing the model to focus on the most relevant features, based on the information provided by other jointly learned tasks.

## Methods

### Software packages and tools

The code developed during this research was implemented as a Python package running on a Linux platform and employs bioinformatics, data analysis, and ML packages. Specifically, the bioinformatics packages include ViennaRNA (v2.4.13) [62], Biopython (v1.72) [63], and NCBI Blast [64]; the data analysis packages include pandas (v0.23.4) [65] and numpy (v1.15.4) [66]; and the ML packages include scikit-learn (v0.20.1) [67] and XGBoost (v0.81) [60].

### Data processing

We acquired eight high-throughput chimeric miRNA–target datasets from four different species: human, mouse, cattle (*Bos taurus*), and worm (*Caenorhabditis elegans*) (Table 1). The details of each dataset are provided in Table 1, including the cell type or developmental stage that were examined and the experimental methods to obtain the data. Five of the datasets (*ca1*, *ce1*, *h1*, *h3*, *m2*) were generated by AGO-CLIP with an extra step to covalently ligate the miRNA and the target RNA. An additional *C. elegans* dataset (*ce2*) contains chimeras recovered from an iCLIP experiment that did not apply an additional ligation step. Two datasets (*h2*, *m1*) were generated by a re-analysis of published mammalian AGO-CLIP data, which also recovered miRNA–target chimeras in libraries in which no ligase was added [21]. The *h2* and *m1* datasets contain chimeras from a mixture of six and three independent experiments, respectively.

We downloaded the datasets’ files from the journals’ websites [18–22]. In addition, we downloaded miRNA sequences from miRBase (releases 17–22) [3], and 3’UTR sequences from the Ensembl Biomart database [68]. We downloaded genomic sequences for *C. elegans* from wormBase [69], and for human and mouse from the UCSC Genome Browser [70]. The datasets are provided in different formats, containing different levels of information about the interactions. Therefore, we developed a processing pipeline to transform the datasets into a standard format and to include the following fields: metadata (interaction ID, interaction source), miRNA name and sequence, target site sequence (the site where the interaction occurred), and, for sites located at the 3’UTRs, the corresponding 3’UTR sequence and the coordinates of the site within it.

We started the pipeline by retrieving the missing miRNA sequences by their name from miRBase (for datasets *ca1, ce2, h3, m2*). Then, we extracted the target sequences (for datasets *ce2, h3, m2*) based on the genomic coordinates. The target sequences are located in various mRNA regions, such as the 5’UTR, the coding sequence, or the 3’UTR. miRNA target sites located at the 3’UTRs of mRNA sequences are considered to be the most functional sites [35, 71]. Therefore, in our analyses, we discarded sites that fall outside the 3’UTRs. Since most datasets do not provide the regions containing the interactions, our next step was to obtain that information. We used Blast [64] to match the target mRNA sequences against the 3’UTRs downloaded from the Ensembl Biomart database. We considered only full-match results. In cases where multiple UTRs exist per gene, we considered the longest UTR. The full 3’UTR sequences were kept for the extraction of flanking site features, as described below. Finally, we took the list of miRNA–target pairs and examined the hybrid structure of the interacting sequences. We applied the ViennaRNA suite (RNAduplex) [62] to calculate the interaction duplex, using the miRNA and the target site sequences. We then classified the duplexes based on their seed type: canonical seed, non-canonical seed, and “other”. Canonical seed interactions are defined as interactions with exact Watson–Crick pairing in positions 2–7 or 3–8 of the miRNA, while non-canonical seed interactions may contain *GU* base-pairs and up to one bulged or mismatched nucleotide at these positions [18]. We kept only canonical and non-canonical seed interactions, and discarded all other interactions from the analysis. Interactions that passed all pipeline stages were designated as positive interactions and were considered for further analysis (Table 8).

### Generation of negative interactions

To generate the negative interactions, we used a synthetic method similar to that described in [35, 72, 73]. For each positive interaction appearing in the dataset, we generated a negative interaction as follows: First, we generated a mock miRNA sequence by randomly shuffling the original sequence until there was, at most, one match in the regions 2–7 and 3–8 between the mock miRNA and any real miRNA of the examined species (according to miRBase). Next, we provided the mock miRNA and the full 3’UTR sequence as inputs to RNAduplex, which is optimized for computing the hybrid structure between a short probe sequence and a long target sequence. We repeated these two steps until the output duplex had either a canonical seed or a non-canonical seed. We managed to generate a negative interaction for each positive interaction, such that, at the end of this process, the datasets were balanced.

### Calculation of miRNA distribution

We counted the occurrence of each miRNA sequence within a dataset and used this information to generate the cumulative distribution function (CDF), shown in Fig. 2. We used the *argmax* function to find the 90% value, which returns the first point in the CDF that is higher than 90%. The seed distribution was calculated by first clustering the miRNA sequences based on their seed sequence (position 2–7), and then following the same steps described above.

### Features

To represent miRNA–target interactions, we used 490 expert-designed features, which are classified into two categories (high level and low level) and five subcategories (Table 9). Four of the subcategories (free energy, mRNA composition, miRNA pairing, and site accessibility) were adopted from [42], while the seed features group was designed during this work. For a full description of the features, see Additional file 4: Table S9.

The *free energy* category includes seven features representing the minimum free energy of the miRNA–mRNA duplex and the mRNA sequence at different regions, including seed, non-seed, site, and flanking regions.

The *mRNA composition* category consists of 62 features that provide information regarding the target mRNA, namely, the distance of the site from the edges of the 3’UTR (two features), 1- and 2-mer sequence composition within the site region (20 features), and 1- and 2-mer sequence composition of the up and down 70nt flanking region (20 features each).

The *miRNA pairing* category consists of 38 features that describe the duplex itself, including information about base-pairs in each location of the miRNA (20 features) and a total count of base-pairs, mismatches, and gaps in the site region (18 features).

The *site accessibility* features were calculated for each 3’UTR sequence containing the seed site, using RNAplfold in the ViennaRNA package [62] with the following parameters: *winsize = 80*, *span = 40*, and *ulength = 10*, as was suggested by previous works [35, 42]. The output of RNAplfold provided, for each nucleotide, the mean probability that regions of lengths 1–10 (ulength), ending at this nucleotide, are unpaired. Of these calculations, we considered only the region that corresponds to the seed region on the target mRNA (p2–p8) with 15 flanking bases to either side (37 bases in total), resulting in $37 \times 10 = 370$ features.

In addition to the above-mentioned features, we designed a new representation for the *seed features*, which describes the base-pairing characteristics of the seed region (positions 1–8 on the miRNA). This new representation includes 13 features: three features describe the number of interactions in [nt1–8, nt2–7, and nt3–8]; three features describe the number of GUs in [nt1–8, nt2–7, and nt3–8]; three features provide information about the number of mismatches (before the first match, inside the seed, and after the last match in the seed region); two features describe the number of bulges (miRNA side and target side); and two features address additional properties (starts with A and index of the first base-pair).

### Splitting of the data into training and testing sets

The appropriate determination of the training and testing sets is crucial for obtaining reliable results. Specifically, the testing set must be sufficiently large, cannot contain samples from the training set, and it needs to be representative of the entire dataset. Accordingly, we implemented a stratified random split algorithm. The algorithm ensures that each miRNA appears in both the training and testing sets at the same proportion as in the original dataset. For example, if a specific miRNA constitutes 10% of the interactions in the original dataset, the algorithm ensures that its proportion in both the training and testing sets is 10%. Within the stratified split, the assignment of the interactions to training (80%) and testing (20%) sets was done randomly according to a random state. The interactions of miRNAs that appeared only once in the dataset were assigned to the testing set. We repeated this process 20 times with different random states, yielding 20 training sets and their corresponding 20 testing sets for each dataset. In addition, for each dataset, we generated five control sets by a fully random algorithm, which does not take into account miRNA distributions. We used these sets as a reference baseline, to assess the influence of the stratified split algorithm on the results (Additional file 1: Section 2).

### Evaluation of different machine-learning methods

To classify miRNA–target interactions, we chose six ML methods that are widely used in the field of computational biology: XGBoost [60], Random Forest (RF), K-nearest neighbors vote (KNN), regularized linear models with Stochastic Gradient Descent (SGD), Support Vector Machine (SVM), and Logistic Regression (LR). We performed the following optimization and learning steps for every combination of (dataset, classifier, data split), altogether yielding 1200 computationally intensive tasks (Eq. 1):

1 $$\begin{aligned} \begin{aligned} optimization \, tasks&= \#classifiers \times \#datasets \times \left( stratified\, splits + control\, splits \right) \\&= 6 \times 8 \times ( 20 + 5 ) \\&= 1200 \end{aligned} \end{aligned}$$

First, we searched for the classifiers’ optimal hyper-parameters. We performed an exhaustive search using sklearn GridSearchCV with a 4-fold cross validation, optimized for accuracy performance. Then, we explored the exhaustive search results and identified the set of parameters that achieved the best accuracy results. We saved the classifier corresponding to this set of parameters and used it to evaluate the accuracy of classification on the testing set. We provide the values of the parameters for the hyper-parameter optimization in Additional file 3 and the mean and standard deviations of the accuracy results (for the 20 stratified splits and the 5 control splits) in the "Results" section and in Additional file 1, respectively.

We continued with the XGBoost classifier to calculate the detailed performance measurements and analyze feature importance. We calculated six widely used performance metrics, including accuracy (ACC), sensitivity (true positive rate, TPR), and specificity (true negative rate, TNR). In addition, we calculated metrics that are widely used for model comparisons, such as the Area Under the Receiver Operating Characteristic Curve (ROC AUC), the Matthews Correlation Coefficient (MCC), and the F1 score (also known as the balanced F-score or F-measure). The description of these metrics and the equations for their calculation are provided in Additional file 1: Section 3 and Equations S1–S5. The means and standard deviations for each measure were calculated on the 20 stratified training–testing splits (Table 5).

### Identification of the top important features

First, we extracted the top important features for each dataset by using the gain metric provided by XGBoost, calculating the mean gain of each feature across the 20 different stratified splits, and sorting the list of features according to the mean gain. We found that the top six features are the most dominant ones (for all datasets) and that the gain score of the other features is lower by an order of magnitude. Therefore, we kept only the top six features of each dataset. Second, to enable comparisons between datasets, we scaled the mean gain scores of each dataset to a range of 0–100 by dividing it by the maximum value and multiplying by 100. Third, we composed a unified list of the top features from all datasets and generated a table that includes the scaled mean gain values for each feature (row) in each dataset (column). Finally, we calculated the mean score for each feature across all datasets (last column in the table) and sorted the table in descending order (see Table 6).

### Calculation of the Kullback–Leibler divergence

The KL divergence is calculated on two probability distribution functions and measures the difference and the distance between them, according to Eq. (2).

2 $$\begin{aligned} D_{KL} \left( P ||Q \right) = \sum _{x\in \chi }{P\left( x \right) log\left( \frac{P\left( x \right) }{Q\left( x \right) } \right) } \end{aligned}$$

We used the KL divergence to measure the pairwise information loss between each two datasets. *P(x)* and *Q(x)* are the miRNA seed distribution functions, as explained in the "Calculation of miRNA distribution" section, above. *Q(x)* is the approximation distribution (calculated from the training set) and *P(x)* is the true distribution (calculated from the testing set). $\chi$ is the union of all the miRNA seeds that appear in both datasets.

### Dimensionality reduction using PCA

The dimensionality reduction algorithm enables the representation of the data in a 2-dimensional scatter plot and facilitates the visual inspection of the data. We performed a dimensional reduction using the PCA algorithm to transform the datasets into 2-dimensional representations. We used the same transformer for all datasets, so as to enable their comparison. We extracted the columns corresponding to the top 16 features that were found as described in "Identification of the top important features" section; we refer to these features as the selected features. Since the datasets are of different sizes, we first oversampled the datasets by a random sampler to bring them to the size of the largest dataset. Then, we concatenated the oversampled datasets together. Next, we standardized the selected features by subtracting the mean and scaling to the unit variance for each feature independently. Finally, we fitted a PCA transformer and applied it to the original datasets (without oversampling), yielding the two-dimensional representation of the datasets on the same vector space. The dimensionality reduction was conducted on the positive experimental interactions only.

### Evaluation of the classification performance between datasets

We evaluated the performance of XGBoost in the classification of interactions derived from a dataset that is different from the dataset it was trained on. We enumerated over all the 56 possible pairs of training and testing datasets: (${train_{i}, test_{j}}$). For each pair, we loaded 20 XGBoost classifiers (corresponding to 20 splits) generated for dataset *i* (as described in "Evaluation of different machine-learning methods" section) and evaluated their performance on the entire dataset *j* (without splitting it). Then, we calculated the mean and the standard deviation of the accuracy results of the 20 tests. A similar analysis was also performed with other examined ML methods.

**Table 1:** Dataset information

Name	Species and cell type/Developmental stage	Experimental method	References
ca1	*B. taurus*, Madin–Darby bovine kidney (MDBK) cells	CLEAR-CLIP	[20]
ce1	*C. elegans*, L3 staged worms	Modified iPAR-CLIP	[21]
ce2	*C. elegans*, Mid-L4 WT (N2) worms	ALG-1 iCLIP endogenous ligation	[22]
h1	Human embryonic kidney293 cells (HEK293)	CLASH	[18]
h2	Human, a mix of 6 datasets	AGO-CLIP endogenous ligation	[21]
h3	Human hepatoma cells (Huh-7.5)	CLEAR-CLIP	[19]
m1	Mouse, a mix of 3 datasets	AGO-CLIP endogenous ligation	[21]
m2	Mouse neuroblastoma N2A cells (ATCC)	CLEAR-CLIP	[19]

**Table 2:** Summary of the data processing pipeline

Dataset	ca1	ce1	ce2	h1	h2	h3	m1	m2
No. of interactions^a^	296,297	3627	4920	18,514	10,567	32,712	1986	130,094
No. of interactions in 3’UTRs	30,534	1704	1206	8507	2039	4634	902	33,100
Final dataset (canonical and non-canonical interactions)	18,204	1176	992	5137	1150	2846	537	17,574

^a^As provided by the original publications

**Table 3:** Composition of miRNA sequences and miRNA seed families within datasets

Dataset	ca1	ce1	ce2	h1	h2	h3	m1	m2
No. of interactions	18,204	1176	992	5137	1150	2846	537	17,574
No. of miRNA sequences	165	68	56	287	140	203	98	417
90% point [miRNA sequences]	49	26	24	99	58	68	49	111
	(29%)	(38%)	(42%)	(34%)	(41%)	(33%)	(50%)	(26%)
No. of seed families	119	46	35	254	133	191	88	343
90% point [seed families]	21	14	13	62	35	42	30	63
	(18%)	(30%)	(37%)	(24%)	(26%)	(22%)	(34%)	(18%)

**Table 4:** Intra-dataset classification accuracy of different machine learning methods

Dataset	XGBoost	RF	KNN	SGD	SVM	LR
ca1	0.937	0.885	0.828	0.797	0.895	0.836
	(0.002)	(0.004)	(0.003)	(0.033)	(0.003)	(0.004)
ce1	0.889	0.833	0.768	0.798	0.841	0.843
	(0.014)	(0.019)	(0.019)	(0.045)	(0.015)	(0.014)
ce2	0.891	0.858	0.768	0.819	0.862	0.847
	(0.016)	(0.018)	(0.019)	(0.034)	(0.012)	(0.016)
h1	0.824	0.769	0.731	0.746	0.795	0.770
	(0.007)	(0.008)	(0.007)	(0.011)	(0.007)	(0.007)
h2	0.904	0.869	0.857	0.860	0.879	0.892
	(0.007)	(0.011)	(0.009)	(0.03)	(0.009)	(0.009)
h3	0.835	0.769	0.744	0.752	0.805	0.795
	(0.007)	(0.009)	(0.009)	(0.034)	(0.007)	(0.010)
m1	0.847	0.795	0.758	0.760	0.819	0.800
	(0.015)	(0.016)	(0.022)	(0.038)	(0.019)	(0.019)
m2	0.900	0.826	0.797	0.798	0.873	0.833
	(0.004)	(0.004)	(0.004)	(0.017)	(0.004)	(0.004)

The cells contain the means and standard deviations (in brackets) of the accuracy results acquired from 20 models that were trained and evaluated on different training-testing dataset splits

**Table 5:** XGBoost performance measurements

Dataset	AUC^a^	ACC ^b^	TPR ^c^	TNR^d^	MCC ^e^	F1 score
ca1	0.983	0.937	0.932	0.943	0.874	0.937
	(0.001)	(0.002)	(0.004)	(0.004)	(0.004)	(0.002)
ce1	0.955	0.889	0.89	0.889	0.779	0.89
	(0.009)	(0.014)	(0.018)	(0.014)	(0.028)	(0.014)
ce2	0.958	0.891	0.884	0.899	0.783	0.89
	(0.012)	(0.016)	(0.02)	(0.019)	(0.032)	(0.017)
h1	0.908	0.824	0.816	0.833	0.649	0.822
	(0.006)	(0.007)	(0.008)	(0.008)	(0.014)	(0.007)
h2	0.972	0.904	0.886	0.924	0.809	0.902
	(0.003)	(0.007)	(0.012)	(0.011)	(0.014)	(0.007)
h3	0.914	0.835	0.823	0.849	0.671	0.832
	(0.004)	(0.007)	(0.011)	(0.009)	(0.014)	(0.008)
m1	0.914	0.847	0.834	0.862	0.695	0.844
	(0.007)	(0.015)	(0.014)	(0.024)	(0.031)	(0.014)
m2	0.963	0.9	0.891	0.909	0.8	0.899
	(0.002)	(0.004)	(0.003)	(0.005)	(0.008)	(0.004)

The cells contain the means and standard deviations (in brackets) acquired from 20 models that were trained and evaluated on different training-testing dataset splits

^a^Area under the receiver operating characteristic curve

^b^Overall accuracy

^c^True Positive Rate (Sensitivity)

^d^True Negative Rate (Specificity)

^e^Matthews correlation coefficient

**Table 6:** Feature importance

Feature/Dataset	ca1	ce1	ce2	h1	h2	h3	m1	m2	Mean
*Number of GU bp within the seed*$^{n}$	100*	87*	95*	29*	40*	100*	28	100*	72
*bp in the 1st nt of the seed*$^{b}$	63*	79	34*	70*	25*	30*	27	85*	52
Number of GU bp within the site$^{n}$	42*	71*	32*	100*	19	53*	35*	28*	48
Proportion of G in mRNA at the site region$^{n}$	12	74*	12	12	36*	33*	100*	37*	39
Duplex minimum free energy$^{n}$	13*	45	11	10	100*	19	35*	52*	36
*Number of bp at location 2–7*$^n$	42*	33	100*	12	18	36*	13	18	34
Proportion of GG in mRNA at the site region$^{n}$	30*	21	10	12	7	30*	79*	26*	27
*bp in the 4th nt of the seed* $^{b}$	8	100*	21	10	11	16	2	12	22
Number of bulges outside the seed$^{n}$	3	60*	6	25*	32*	9	9	8	19
*bp in the 2nd nt of the seed * $^{b}$	8	42	37*	7	11	13	15	6	17
*bp in the 5th nt of the seed* $^{b}$	12	27	14	14	6	15	29*	12	16
*Number of GC bp within the seed* $^{n}$	7	22	24*	18*	12	13	11	12	15
Number of GC bp outside the seed$^{n}$	4	27	11	10	27*	8	6	5	12
Accessibility (nt = 21, len = 10)$^{n}$	9	19	7	6	25	7	12	7	11
minimum free energy of the target site + 50nt flanking regions$^n$	8	11	6	7	8	11	36*	6	11
*Number of mismatches inside the seed* $^n$	4	3	15	19*	0	13	2	9	8

The table shows 16 features representing the union of the top 6 features of each dataset, along with their gain values which were computed by XGBoost. The features are ordered by their mean gain, scaled to the range of (0, 100), across all datasets. For the unscaled version of the table, see Additional file 1: Table S5

*Belongs to the top 6 features of the dataset

$$^b$$Boolean feature

$$^n$$Numeric feature

**Table 7:** Estimated divergence time [MYA] between species in our study

	Mouse	Cattle	*C. elegans*
Human	90	96	797
Mouse		96	797
Cattle			797

Each cell represents the time since the pair of species from the corresponding row and column diverged from their common ancestor (*Source*: [74])

**Table 8:** Data processing pipeline

Source	[18]	[21]	[20]	[22]	[19]
Datasets	h1	ce1, h2, m1	ca1	ce2	h3, m2
miRNA sequence	$\checkmark$	$\checkmark$	miRBASE	miRBASE	miRBASE
Target sequence	$\checkmark$	$\checkmark$	$\checkmark$	Wormbase	UCSC genome browser
Site region	Ensembl Biomart + Blast				
Duplex structure	Vienna RNAduplex				
Seed filter	Canonical and non-canonical seeds only				

The table describes the set of actions required to transform the datasets into a uniform format to serve as input for further data analysis and machine learning experiments. The check-mark sign ($\checkmark$) represents a piece of information taken directly from the paper without additional calculations

**Table 9:** Feature categories that are used to represent miRNA–target interactions

Category	No. of features	Description	Group
Seed features	13	Seed composition and properties	High-level
Free energy	7	Free energy of the duplex and the mRNA at different regions	High-level
mRNA composition	62	mRNA composition in the site and flanking regions	High-level
miRNA pairing	38	Binding information at each miRNA position and across the miRNA–target duplex	Low-level
Site accessibility	370	Unpaired probabilities of each base	Low-level
Total	490		

## Supplementary Information

**Additional file 1**. 1. Review of Machine-Learning (ML) based methods; 2. Training and testing random dataset split; 3. Description of the classification performance metrics;  Supplemental Figure S1 to S7;  Supplemental Tables S1 to S7; Equations S1 to S5.

**Additional file 2**. Table S8. Feature importance.

**Additional file 3**. Grid search params.yaml.

**Additional file 4**. Table S9. Features and their definition.

## Data Availability

The datasets used and/or analyzed during the current study are available from the corresponding author upon reasonable request.

## Abbreviations

ACC: Accuracy
AUC: Area under the curve
BP: Base-pair
CLASH: Cross-linking, ligation and sequencing of hybrids
CLIP: Cross-linking immunoprecipitation
KL: Kullback–Leibler
LR: Logistic Regression
MCC: Matthews correlation coefficient
miRISC: miRNA-induced silencing complex
ML: Machine learning
PCA: Principal component analysis
RF: Random Forest (RF)
SGD: Stochastic Gradient Descent
SVM: Support vector machine
TNR: True Negative Rate
TPR: True Positive Rate
UTR: Untranslated region

## References

[1] Finnegan EF, Pasquinelli AE (2013). Microrna biogenesis: regulating the regulators. Crit Rev Biochem Mol Biol.

[2] Huntzinger E, Izaurralde E (2011). Gene silencing by microRNAS: contributions of translational repression and MRNA decay. Nat Rev Genet.

[3] Kozomara A, Griffiths-Jones S (2013). miRBASE: annotating high confidence microRNAS using deep sequencing data. Nucleic Acids Res.

[4] Rupaimoole R, Slack FJ (2017). Microrna therapeutics: towards a new era for the management of cancer and other diseases. Nat Rev Drug Discov.

[5] Li J, Zhang Y (2019). Current experimental strategies for intracellular target identification of microrna. ExRNA.

[6] Martinez-Sanchez A, Murphy CL (2013). Microrna target identification–experimental approaches. Biology.

[7] Thomas M, Lieberman J, Lal A (2010). Desperately seeking microRNA targets. Nat Struct Mol Biol.

[8] Fabian MR, Sonenberg N, Filipowicz W (2010). Regulation of MRNA translation and stability by microRNAS. Annu Rev Biochem.

[9] Chi SW, Zang JB, Mele A, Darnell RB (2009). Argonaute hits-clip decodes microRNA–MRNA interaction maps. Nature.

[10] Zisoulis DG, Lovci MT, Wilbert ML, Hutt KR, Liang TY, Pasquinelli AE, Yeo GW (2010). Comprehensive discovery of endogenous argonaute binding sites in *Caenorhabditis elegans*. Nat Struct Mol Biol.

[11] Hafner M, Landthaler M, Burger L, Khorshid M, Hausser J, Berninger P, Rothballer A, Ascano M, Jungkamp A-C, Munschauer M (2010). Transcriptome-wide identification of RNA-binding protein and microRNA target sites by par-clip. Cell.

[12] Wang T, Xiao G, Chu Y, Zhang MQ, Corey DR, Xie Y (2015). Design and bioinformatics analysis of genome-wide clip experiments. Nucleic Acids Res.

[13] Uhl M, Houwaart T, Corrado G, Wright PR, Backofen R (2017). Computational analysis of CLIP-seq data. Methods.

[14] Majoros WH, Lekprasert P, Mukherjee N, Skalsky RL, Corcoran DL, Cullen BR, Ohler U (2013). Microrna target site identification by integrating sequence and binding information. Nat Methods.

[15] Reczko M, Maragkakis M, Alexiou P, Grosse I, Hatzigeorgiou AG (2012). Functional microRNA targets in protein coding sequences. Bioinformatics.

[16] Liu C, Mallick B, Long D, Rennie WA, Wolenc A, Carmack CS, Ding Y (2013). Clip-based prediction of mammalian microRNA binding sites. Nucleic Acids Res.

[17] Khorshid M, Hausser J, Zavolan M, Van Nimwegen E (2013). A biophysical miRNA–mRNA interaction model infers canonical and noncanonical targets. Nat Methods.

[18] Helwak A, Kudla G, Dudnakova T, Tollervey D (2013). Mapping the human miRNA interactome by clash reveals frequent noncanonical binding. Cell.

[19] Moore MJ, Scheel TK, Luna JM, Park CY, Fak JJ, Nishiuchi E, Rice CM, Darnell RB (2015). miRNA-target chimeras reveal miRNA 3’-end pairing as a major determinant of argonaute target specificity. Nat Commun.

[20] Scheel TK, Moore MJ, Luna JM, Nishiuchi E, Fak J, Darnell RB, Rice CM (2017). Global mapping of miRNA-target interactions in cattle (Bos taurus). Sci Rep.

[21] Grosswendt S, Filipchyk A, Manzano M, Klironomos F, Schilling M, Herzog M, Gottwein E, Rajewsky N (2014). Unambiguous identification of miRNA: target site interactions by different types of ligation reactions. Mol Cell.

[22] Broughton JP, Lovci MT, Huang JL, Yeo GW, Pasquinelli AE (2016). Pairing beyond the seed supports microRNA targeting specificity. Mol Cell.

[23] Krüger J, Rehmsmeier M (2006). RNAhybrid: microRNA target prediction easy, fast and flexible. Nucleic Acids Res.

[24] Enright AJ, John B, Gaul U, Tuschl T, Sander C, Marks DS (2003). Microrna targets in drosophila. Genome Biol.

[25] Lewis BP, Burge CB, Bartel DP (2005). Conserved seed pairing, often flanked by adenosines, indicates that thousands of human genes are microRNA targets. Cell.

[26] Kertesz M, Iovino N, Unnerstall U, Gaul U, Segal E (2007). The role of site accessibility in microRNA target recognition. Nat Genet.

[27] Pinzón N, Li B, Martinez L, Sergeeva A, Presumey J, Apparailly F, Seitz H (2017). microRNA target prediction programs predict many false positives. Genome Res.

[28] Oliveira AC, Bovolenta LA, Nachtigall PG, Herkenhoff ME, Lemke N, Pinhal D (2017). Combining results from distinct microRNA target prediction tools enhances the performance of analyses. Front Genet.

[29] Fridrich A, Hazan Y, Moran Y (2019). Too many false targets for microRNAS: challenges and pitfalls in prediction of miRNA targets and their gene ontology in model and non-model organisms. BioEssays.

[30] Min H, Yoon S (2010). Got target? Computational methods for microRNA target prediction and their extension. Exp Mol Med.

[31] Xiao F, Zuo Z, Cai G, Kang S, Gao X, Li T (2009). miRecords: an integrated resource for microRNA-target interactions. Nucleic Acids Res.

[32] Chou C-H, Chang N-W, Shrestha S, Hsu S-D, Lin Y-L, Lee W-H, Yang C-D, Hong H-C, Wei T-Y, Tu S-J (2016). miRTarBase 2016: updates to the experimentally validated miRNA–target interactions database. Nucleic Acids Res.

[33] Liu H, Yue D, Chen Y, Gao S-J, Huang Y (2010). Improving performance of mammalian microRNA target prediction. BMC Bioinform.

[34] Yu S, Kim J, Min H, Yoon S (2014). Ensemble learning can significantly improve human microRNA target prediction. Methods.

[35] Menor M, Ching T, Zhu X, Garmire D, Garmire LX (2014). mirMark: a site-level and UTR-level classifier for miRNA target prediction. Genome Biol.

[36] Cheng S, Guo M, Wang C, Liu X, Liu Y, Wu X (2015). MiRTDL: a deep learning approach for miRNA target prediction. IEEE/ACM Trans Comput Biol Bioinf.

[37] Lee B, Baek J, Park S, Yoon S. deepTarget: end-to-end learning framework for microRNA target prediction using deep recurrent neural networks. In: Proceedings of the 7th ACM international conference on bioinformatics, computational biology, and health informatics. 2016. p. 434–42.

[38] Jiang H, Wang J, Li M, Lan W, Wu F-X, Pan Y (2018). miRTRS: a recommendation algorithm for predicting miRNA targets. IEEE/ACM Trans Comput Biol Bioinf.

[39] Lu Y, Leslie CS (2016). Learning to predict miRNA–mRNA interactions from AGO CLIP sequencing and clash data. PLoS Comput Biol.

[40] Ding J, Li X, Hu H (2016). TarPmiR: a new approach for microRNA target site prediction. Bioinformatics.

[41] Pla A, Zhong X, Rayner S (2018). miRAW: a deep learning-based approach to predict microRNA targets by analyzing whole microRNA transcripts. PLoS Comput Biol.

[42] Wen M, Cong P, Zhang Z, Lu H, Li T (2018). DeepMirTar: a deep-learning approach for predicting human miRNA targets. Bioinformatics.

[43] Paker A, Oğul H. mirLSTM: a deep sequential approach to microRNA target binding site prediction. In: International conference on database and expert systems applications. Springer; 2019. p. 38–44.

[44] Wang X (2016). Improving microRNA target prediction by modeling with unambiguously identified microRNA-target pairs from clip-ligation studies. Bioinformatics.

[45] Liu W, Wang X (2019). Prediction of functional microRNA targets by integrative modeling of microRNA binding and target expression data. Genome Biol.

[46] Dweep H, Gretz N (2015). miRWALK 2.0: a comprehensive atlas of microRNA–target interactions. Nat Methods.

[47] Chang L, Zhou G, Soufan O, Xia J (2020). miRNet 2.0: network-based visual analytics for miRNA functional analysis and systems biology. Nucleic Acids Res.

[48] Chen Y, Wang X (2020). miRDB: an online database for prediction of functional microRNA targets. Nucleic Acids Res.

[49] Bartel DP (2009). microRNAS: target recognition and regulatory functions. Cell.

[50] Schirle NT, Sheu-Gruttadauria J, MacRae IJ (2014). Structural basis for microRNA targeting. Science.

[51] Slack FJ, Basson M, Liu Z, Ambros V, Horvitz HR, Ruvkun G (2000). The lin-41 RBCC gene acts in the *C. elegans* heterochronic pathway between the let-7 regulatory RNA and the LIN-29 transcription factor. Mol Cell.

[52] Vella MC, Choi E-Y, Lin S-Y, Reinert K, Slack FJ (2004). The *C. elegans* microRNA let-7 binds to imperfect let-7 complementary sites from the lin-41 3’ utr. Genes Dev.

[53] Chi SW, Hannon GJ, Darnell RB (2012). An alternative mode of microRNA target recognition. Nat Struct Mol Biol.

[54] Agarwal V, Bell GW, Nam J-W, Bartel DP (2015). Predicting effective microRNA target sites in mammalian mRNAs. eLife.

[55] Soriano A, Masanas M, Boloix A, Masiá N, París-Coderch L, Piskareva O, Jiménez C, Henrich K-O, Roma J, Westermann F (2019). Functional high-throughput screening reveals miR-323a-5p and miR-342-5p as new tumor-suppressive microRNA for neuroblastoma. Cell Mol Life Sci.

[56] Brennecke J, Stark A, Russell RB, Cohen SM (2005). Principles of microRNA–target recognition. PLoS Biol.

[57] Grimson A, Farh KK-H, Johnston WK, Garrett-Engele P, Lim LP, Bartel DP (2007). microRNA targeting specificity in mammals: determinants beyond seed pairing. Mol Cell.

[58] Blum AL, Langley P (1997). Selection of relevant features and examples in machine learning. Artif Intell.

[59] Lecun Y. The mnist database of handwritten digits. http://yann.lecun.com/exdb/mnist/

[60] Chen T, Guestrin C. XGBoost: a scalable tree boosting system, p. 785–794 (2016). 10.1145/2939672.2939785.

[61] Nielsen D. Tree boosting with xgboost-why does xgboost win“every” machine learning competition? Master’s thesis, NTNU; 2016.

[62] Lorenz R, Bernhart SH, Zu Siederdissen CH, Tafer H, Flamm C, Stadler PF, Hofacker IL (2011). Viennarna package 2.0. Algorithms Mol Biol.

[63] Cock PJ, Antao T, Chang JT, Chapman BA, Cox CJ, Dalke A, Friedberg I, Hamelryck T, Kauff F, Wilczynski B (2009). Biopython: freely available Python tools for computational molecular biology and bioinformatics. Bioinformatics.

[64] Altschul SF, Gish W, Miller W, Myers EW, Lipman DJ (1990). Basic local alignment search tool. J Mol Biol.

[65] McKinney W,et al. Data structures for statistical computing in Python. In: Proceedings of the 9th Python in science conference, Austin, TX, vol. 445, 2010; p. 51–56.

[66] Oliphant TE. A guide to NumPy, vol. 1. Trelgol Publishing; 2006.

[67] Pedregosa F, Varoquaux G, Gramfort A, Michel V, Thirion B, Grisel O, Blondel M, Prettenhofer P, Weiss R, Dubourg V (2011). Scikit-learn: machine learning in Python. J Mach Learn Res.

[68] Smedley D, Haider S, Durinck S, Pandini L, Provero P, Allen J, Arnaiz O, Awedh MH, Baldock R, Barbiera G (2015). The BioMart community portal: an innovative alternative to large, centralized data repositories. Nucleic Acids Res.

[69] Lee RYN, Howe KL, Harris TW, Arnaboldi V, Cain S, Chan J, Chen WJ, Davis P, Gao S, Grove C (2017). Wormbase 2017: molting into a new stage. Nucleic Acids Res.

[70] Karolchik D, Hinrichs AS, Furey TS, Roskin KM, Sugnet CW, Haussler D, Kent WJ (2004). The UCSC Table Browser data retrieval tool. Nucleic Acids Res.

[71] Baek D, Villén J, Shin C, Camargo FD, Gygi SP, Bartel DP (2008). The impact of microRNAS on protein output. Nature.

[72] John B, Enright AJ, Aravin A, Tuschl T, Sander C, Marks DS (2004). Human microRNA targets. PLoS Biol.

[73] Maragkakis M, Alexiou P, Papadopoulos GL, Reczko M, Dalamagas T, Giannopoulos G, Goumas G, Koukis E, Kourtis K, Simossis VA (2009). Accurate microRNA target prediction correlates with protein repression levels. BMC Bioinform.

[74] Kumar S, Stecher G, Suleski M, Hedges SB (2017). TimeTree: a resource for timelines, timetrees, and divergence times. Mol Biol Evol.

