# Supplementary material for: Comprehensive machine-learning-based analysis of microRNA–target interactions reveals variable transferability of interaction rules across species
Source: BMC Bioinformatics. 2021 May 24;22:264. doi: 10.1186/s12859-021-04164-x (PMC8146624; doi:10.1186/s12859-021-04164-x)
Supplement: Supplementary file 1 — Additional file 1. 1. Review of Machine-Learning (ML) based methods; 2. Training and testing random dataset split; 3. Description of the classification performance metrics; Supplemental Figure S1 to S7; Supplemental Tables S1 to S7; Equations S1 to S5. [file 12859_2021_4164_MOESM1_ESM.pdf]

Comprehensive machine-learning-based analysis of microRNA–target interactions reveals variable transferability of interaction rules across species

Supplementary Material

Gilad Ben Or and Isana Veksler-Lublinsky

1 REVIEW OF MACHINE-LEARNING BASED METHODS

Several studies previously utilized chimeric miRNA–target datasets to build and evaluate machine-learning (ML)-based miRNA–target prediction methods. These methods differ in several aspects, including the ML approach and the features used, the choice of datasets for training and testing, the inclusion or exclusion of non-canonical interactions from the training or testing sets, and the generation of negative data (Table S1). Below, we provide a summary of some of these methods, focusing on these aspects.

1.1 *Machine-learning methods and features*

The method known as chimiRic[8] combines two support vector machine (SVM) classifiers, one to predict miRNA–mRNA duplexes and the other to learn AGO binding preferences. The features used by this method are designed to describe the structure of the duplex (e.g., binding information at each miRNA position[6], positions of loop opening and extension), UTR local sequence context, and global positional context.

TarPmiR[3] is a Random-Forest-based approach that provides the probability that a candidate target site is a true target site. It integrates six conventional features (e.g., folding energy and seed-matching) and seven new features (e.g., largest consecutive pairings).

DeepMirTar[14] is based on a stacked de-noising auto-encoder deep learning method (SdA) and uses 750 different features to describe the interactions (including raw-data-level and expert-designed high-level and low-level features). These features capture the information about the seed match, sequence composition, free energy, site accessibility, conservation, and hot-encoding of miRNAs and their target sites.

miRAW[11] relies on deep artificial neural networks (ANNs) to automatically learn the relevant features describing miRNA–gene interactions to predict miRNA targets. This method works with raw input data and makes no assumptions about suitable input descriptors.

mirLSTM[10] is a deep-learning approach that is based on Long Short Term Memory (LSTM). The method captures only the information encoded in the duplex that is formed between the miRNA and its relevant binding site, which is reduced into a vector over a five-letter alphabet to express four possible base-pairs (*AU,UA,GC,CG*) and one letter for all the remaining combinations (including *GU*, bulge, and mismatch). These vectors are converted into embedded words to find the relationship between similar sequences, using Euclidean distance. Then, the embedded words are fed into the first layer of the LSTM architecture.

MirTarget[13] is based on SVM and uses 50 features that include nucleotide composition, accessibility of the target site,

seed conservation, seed base-pairing stability, and target site location. In a subsequent study[7], the model was refined with 96 features.

1.2 *Training and testing datasets*

All the above-mentioned studies trained and tested their models on a dataset of chimeric interactions from human cells generated with the CLASH method[5], referred to as *h1* in the main manuscript. In some of the studies, the dataset was filtered based on the location of the sites, seed-pairing pattern, or functional evidence; in other studies, it was complemented with additional interactions from other experiments.

DeepMirTar[14] and mirLSTM[10] filtered this dataset to include only canonical and non-canonical sites that are located at the 3'UTRs. They complemented this dataset with an additional small number of interactions retrieved from miRecords[15]. TarPmir[3] used all the interactions available from the human CLASH dataset *h1*. chimiRic[8] was trained on a combination of the human CLASH dataset and seed-containing sites from AGO-CLIP data. The model was tested on specific miRNA families by excluding their interactions from the training set. For miRAW[11], the training set was built by intersecting the human CLASH, AGO-CLIP, and TargetScanHuman[1] datasets with mirTarBase[2] and Diana TarBase[12] to include only validated functional interactions. The initial evaluation was performed on experimentally verified miRNA–targets that were excluded from the training set. miRTarget[13] was trained and tested on human CLASH data combined with chimeras generated by endogenous ligation in human AGO-CLIP experiments[4]. miRTarget v4[7] combined human chimeras with miRNA overexpression data to identify common features that are characteristic of both miRNA binding and target downregulation.

1.3 *Independent testing datasets*

For additional independent testing, the above-mentioned methods employed various datasets, which were not necessarily derived from ligation-based experiments. DeepMirTar[14] was tested on a human PAR-CLIP dataset. mirLSTM[10] was tested on a small experimental dataset. TarPmir[3] was tested on two PAR-CLIP human datasets and a HITS-CLIP dataset from a mouse. chimiRic's[8] model was tested on chimeric interactions from *C. elegans*[4] and mouse[9]. miRAW[11] performed additional evaluations on microarray datasets reporting mRNA changes after transfecting miRNAs into HeLa cells. miRTarget[13] and miRTarget v4[7] tested their model on mRNA microarray datasets from miRNA knockdown experiments. miRTarget v4 performed additional testing on HITS-CLIP data from the mouse brain.

1.4 *Negative datasets*

For negative datasets, DeepMirTar[14] and mirLSTM[10] used mock miRNAs to generate a negative interaction for each positive one. TarPmir[3] generated negative target sites on positive mRNAs, such that they do not overlap with positive sites and their nucleotide composition is similar to the positive sites. miRAW built a negative dataset based upon experimentally verified data. chimiRic[8] generated a negative set from canonical miRNA seed matches that are not AGO-bound based on CLIP data, together with (miRNA, site) pairs where an AGO-bound site is paired with an incorrect miRNA based on CLASH chimeras. miRTarget[13] and miRTarget v4[7] generated negative examples based on CLIP data, selecting non-target sites based on a set of criteria, e.g., no overlap with a positive site, detectable expression of the transcript based on microarrays, or the existence of a perfect seed match to one of the miRNAs expressed in the cells.

2 TRAINING AND TESTING RANDOM DATASET SPLIT

The procedure of splitting the data into training and testing sets has a crucial role in the evaluation of machine-learning models. In the miRNA–target prediction task, there is no pre-defined split to training and testing sets as is common in other fields. In this work, we used a stratified training–testing split that ensures the same distribution of miRNA sequences in both the training and the testing sets. To assess how this type of split affects the classifier performance, we repeated the analysis with the XGBoost classifier, but this time used a random split strategy (control split) to generate the training and testing sets. Our results showed that there is almost no difference between the results achieved with the stratified and the control split methods. As with the stratified split method, we obtained very low standard

deviation values, confirming that the split step did not bias our results. The accuracy results for the control splits are provided in Table S3.

3 DESCRIPTION OF THE CLASSIFICATION PERFORMANCE METRICS

The following metrics were used for an in-depth performance analysis of the XGBoost classifier:

AUC - The area under the curve is a performance measurement for classification problems at different threshold settings. The AUC provides information on the capability of a model to differentiate between classes; AUC values range from 0 to 1, with 1 indicating a model with perfect predictions.

TPR and TNR - The true positive rate (Equation S2) and the true negative rate (Equation S3) are the percentages of actual positive or negative results, respectively, that are correctly identified. For ideal classifiers, these metrics are close to 1.

MCC - the Matthews correlation coefficient (Equation S4) measures the quality of classifications; a coefficient of +1 indicates a perfect prediction, 0 an average random prediction, and -1 an inverse prediction.

F1 score - F1 score (Equation S5) is the average of the precision metric (the proportion of correct positive classifications) and the recall metric (the proportion of positives that were correctly classified), with a value of 1 indicating the best performance and 0 indicating the worst performance.

Figure S1. Classification of the negative miRNA–target duplexes, based on their base-pairing patterns

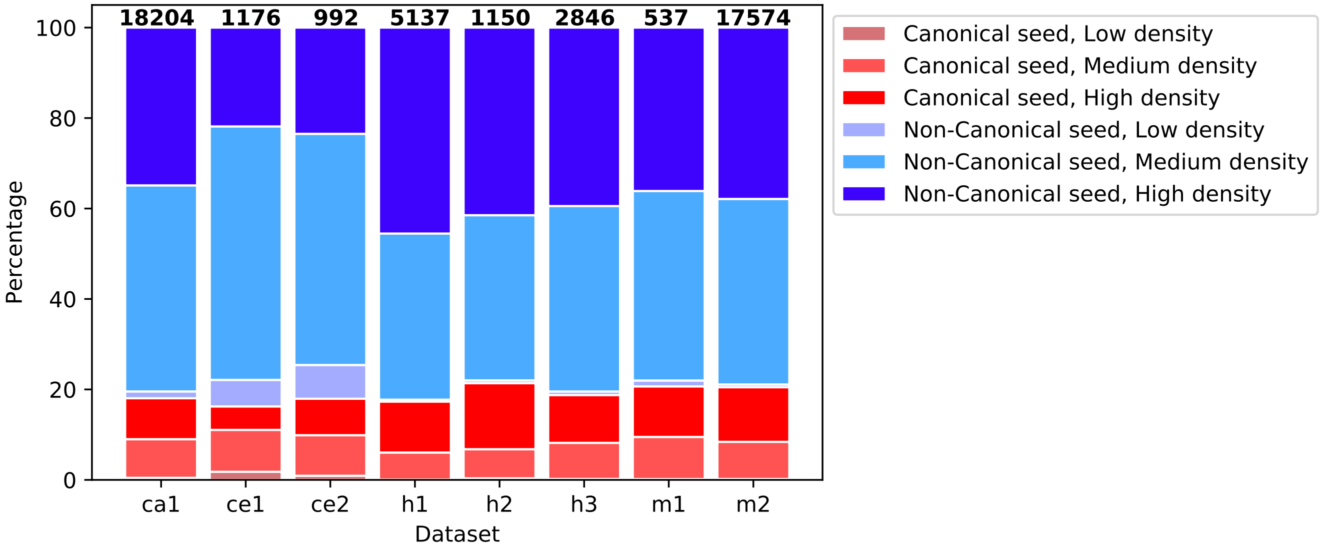

Distribution of miRNA–target duplexes across six classes according to the seed type (canonical or non-canonical) and the base-pairing density (low: $<$than 11 bp, medium: 11–16 bp, or high: more than$>$16bp). The number above each bar indicates the total number of interactions.

Figure S2. Cross-dataset classification results of xGBoost using 16 features

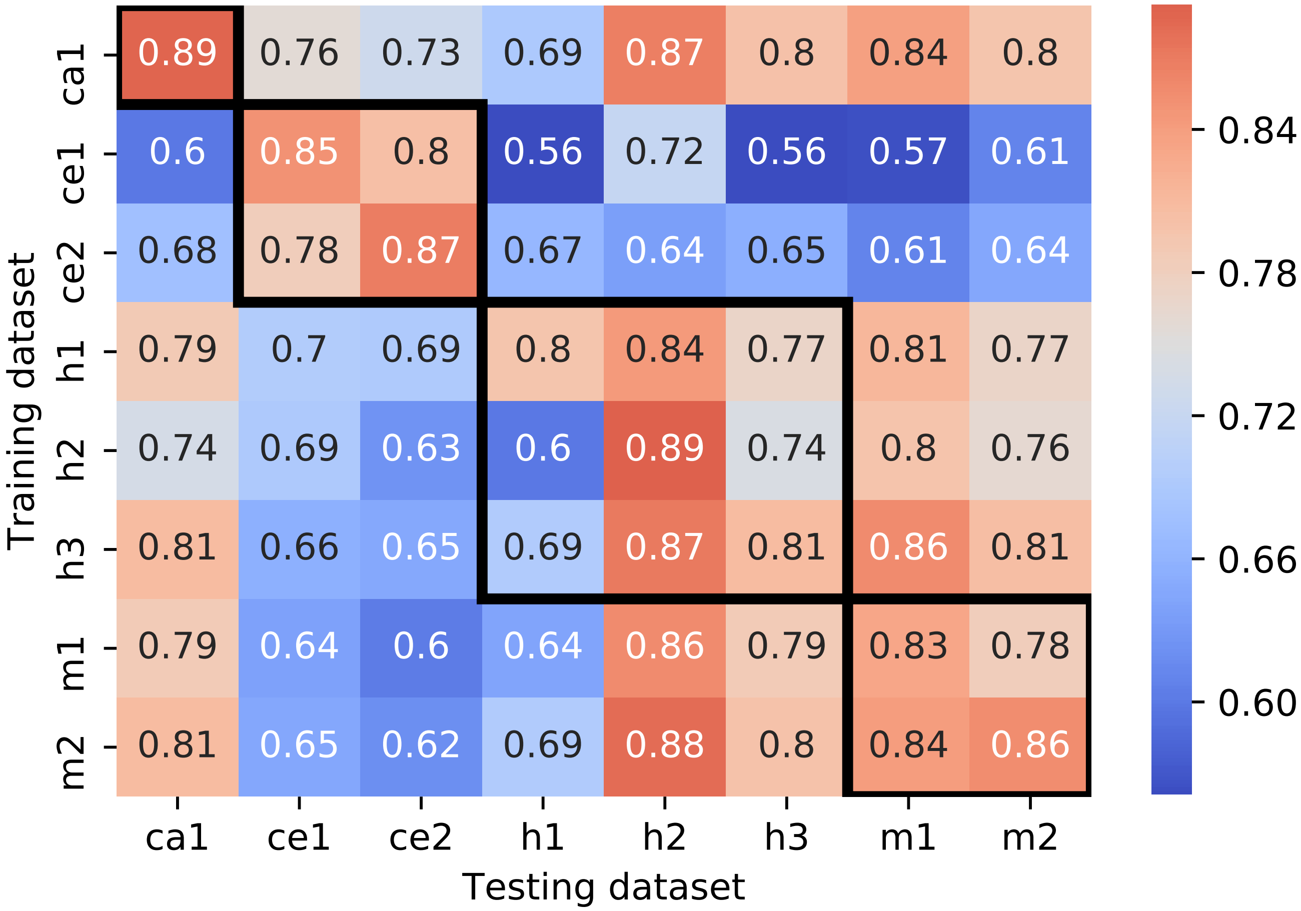

Each cell *(i,j)* represents the mean accuracy of the 20 xGBoost classifiers that were trained on dataset *i* and tested on dataset *j ACC(i, j)* using 16 features found in section *Top important features of each dataset*. The black frames indicate the results of dataset pairs originating from the same species.

Figure S3. Cross-dataset classification results of LogisticRegression with using all features

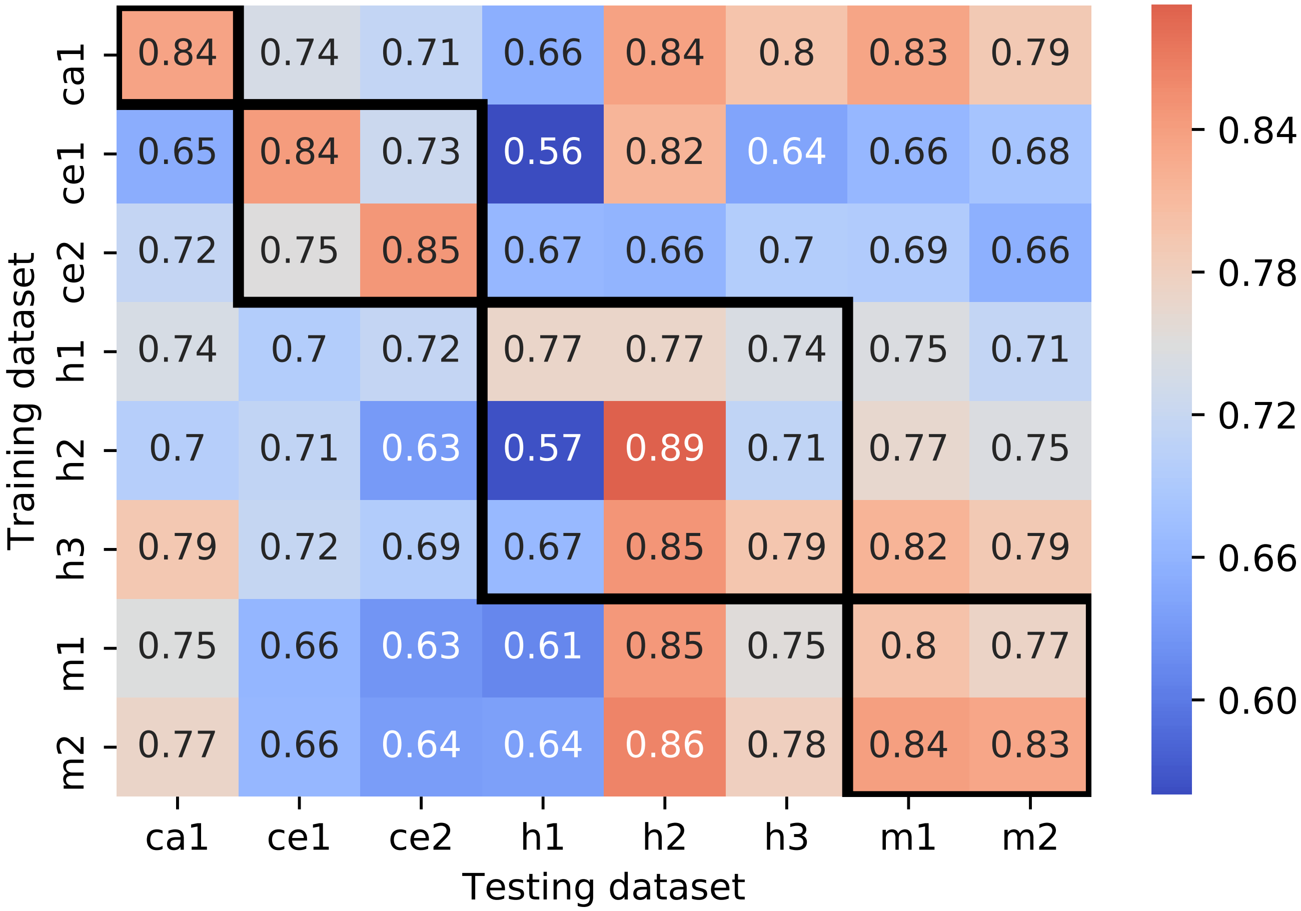

Each cell *(i,j)* represents the mean accuracy of the 20 LogisticRegression classifiers that were trained on dataset *i* and tested on dataset *j* (ACC(i, j)). The black frames indicate the results of dataset pairs originating from the same species.

Figure S4. Cross-dataset classification results of RandomForest using all features

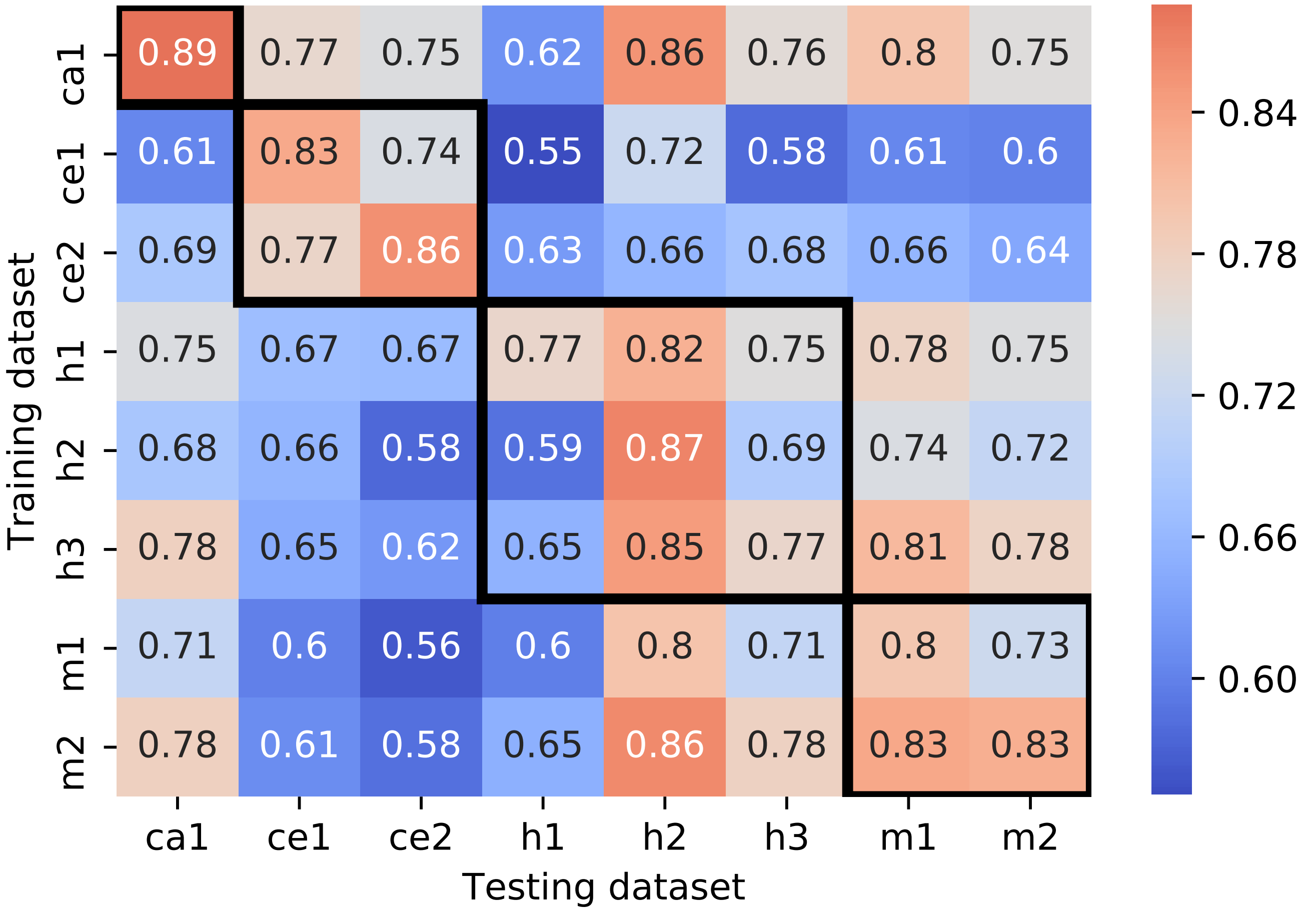

Each cell *(i,j)* represents the mean accuracy of the 20 RandomForest classifiers that were trained on dataset *i* and tested on dataset *j* (ACC(i, j)). The black frames indicate the results of dataset pairs originating from the same species.

Figure S5. Cross-dataset classification results of SGD using all features

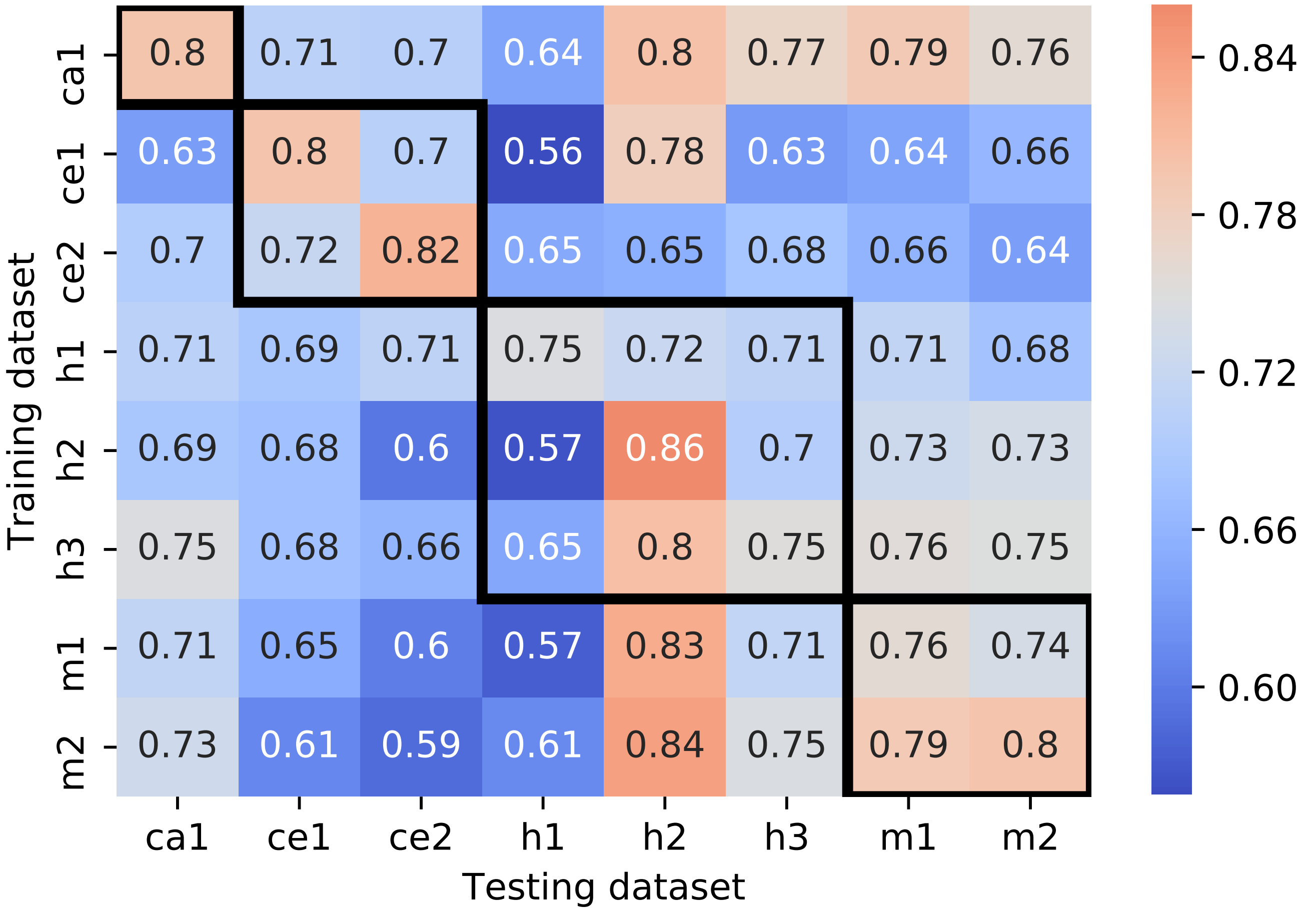

Each cell *(i,j)* represents the mean accuracy of the 20 SGD classifiers that were trained on dataset *i* and tested on dataset *j* (ACC(i, j)). The black frames indicate the results of dataset pairs originating from the same species.

Figure S6. Cross-dataset classification results of SVM using all features

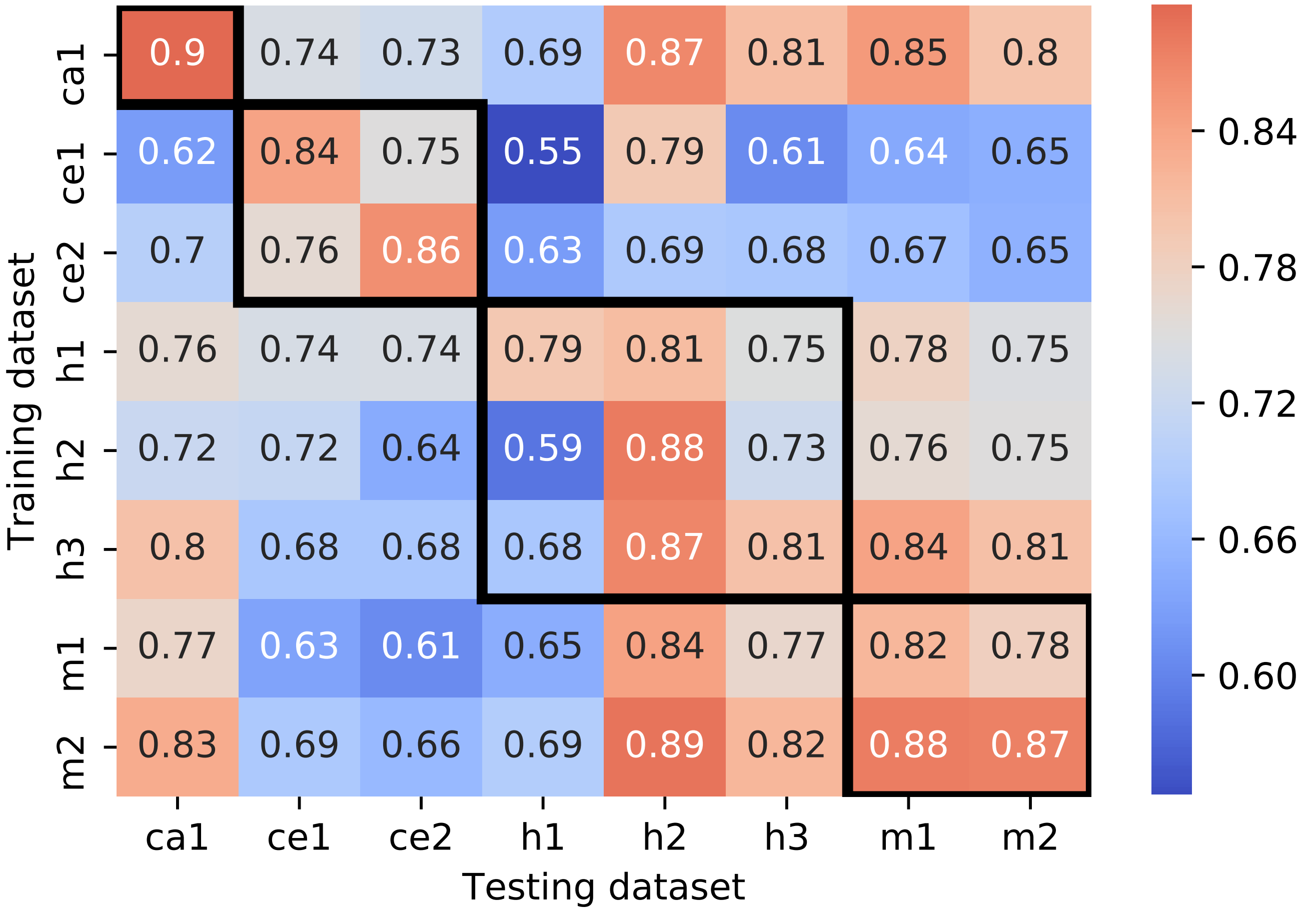

Each cell *(i,j)* represents the mean accuracy of the 20 SVM classifiers that were trained on dataset *i* and tested on dataset *j* (ACC(i, j)). The black frames indicate the results of dataset pairs originating from the same species.

Figure S7. Cross-dataset classification results of KNeighbors using all features

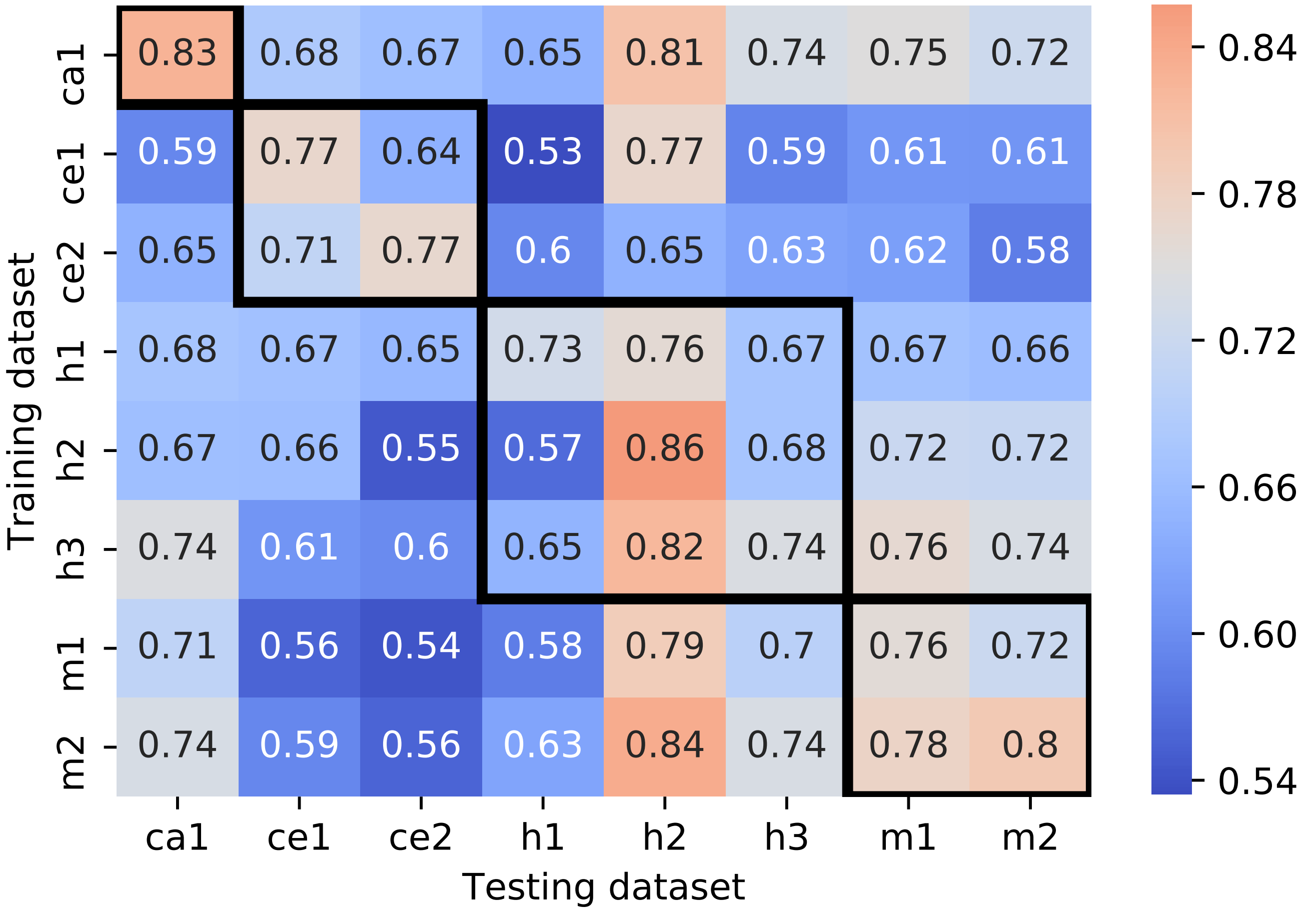

Each cell *(i,j)* represents the mean accuracy of the 20 KNeighbors classifiers that were trained on dataset *i* and tested on dataset *j* (ACC(i, j)). The black frames indicate the results of dataset pairs originating from the same species.

Table S1. A summary of machine-learning based methods that utilized chimeric miRNA–target datasets in their models.

Tool	ML method	Datasets used for training/testing	Independent Dataset	Negative interactions	Features
chimiRic[8]	SVM	Human (CLASH, AGO-CLIP)	Chimeras from Mouse and *C.elegans*	Seed matching non-CLIP sites	Small
TarPmiR[3]	Random Forest	Human (CLASH)	Human (PAR-CLIP), Mouse (HITS-CLIP)	Negative target sites, on positive mRNAs	13
DeepMirTar[14]	Deep learning	Human (CLASH) + mirRecords	Human (PAR-CLIP)	Mock miRNAs	750
miRAW[11]	Deep learning	Human (intersection of: CLASH, CLIP, TargetScan with Diana TarBase, mirTarBase)	Human (microarray)	Experimental data	Raw sequence
mirLSTM[10]	Deep learning	Human (CLASH) + mirRecords	Experimental	Mock miRNAs	Raw sequence
mirTarget[13]	SVM	Human (CLASH, AGO-CLIP)	Human(microarrays)	Seed matching non-CLIP sites on expressed mRNAs	50
mirTarget v4[7]	SVM	Human (Intersection of CLASH and microarrays)	Mouse (HITS-CLIP), Human (microarrays)	Seed matching non-CLIP sites on expressed mRNAs	96

Table S2. The performance accuracy of different tools (left) and machine learning methods (right), on human dataset referred to as *h1* in the main manuscript, taken from deepMirTar [14].

Methods	ACC
Miranda	0.6592
RNAhybrid	0.6988
PITA	0.4981
TargetScan v7.0a	0.5801
TarPmiR	0.7446
DeepMirTar (SdA)	0.9348

Machine learning alg.	ACC
DT (Decision Tree)	0.8139 (0.0137)
BNB (Bernoulli Naïve Bayes)	0.7570 (0.0098)
LR (Logistic Regression)	0.8491 (0.0117)
RF (Random Forest)	0.8811 (0.0090)
MLP (Multi-Layer Perceptron)	0.8990 (0.0099)
CNN-1D (Convolutional Neural Network)	0.8886 (0.0145)
CNN-2D (Convolutional Neural Network)	0.8765 (0.0169)

The right table contains the means and the standard deviations (in brackets) acquired from 20 models that were built based on different random splits of the dataset to training/validation/testing.

Table S3. Intra-dataset classification accuracy of xGBoost when applied to random (control) training-testing dataset splits

Dataset	Accuracy
ca1	0.938 (0.004)
ce1	0.891 (0.013)
ce2	0.89 0(0.012)
h1	0.832 (0.006)
h2	0.920 (0.013)
h3	0.849 (0.008)
m1	0.848 (0.011)
m2	0.897 (0.006)

The cells contain the means and standard deviations (in brackets) acquired from 5 models that were trained and evaluated on different random dataset splits.

Table S4. Intra-dataset classification accuracy of xGBoost classifier trained on an extended 580 features set (including raw-data-level features)

Dataset	Accuracy
ca1	0.985 (0.001)
ce1	0.951 (0.006)
ce2	0.959 (0.008)
h1	0.93 (0.006)
h2	0.922 (0.008)
h2	0.908 (0.007)
m1	0.877 (0.014)
m2	0.956 (0.002)

The cells contain the means and standard deviations (in brackets) values acquired from 20 models that were trained and evaluated on different training-testing stratified dataset splits.

Table S5. Feature importance

**Feature/Dataset**	**ca1**	**ce1**	**ce2**	**h1**	**h2**	**h3**	**m1**	**m2**	**mean**
**Number of GU bp within the seed**	225	25	51	25	23	77	12	172	76
**bp in the 1st nt of the seed**	142	23	18	63	14	23	11	147	55
Number of GU bp within the site	94	20	17	89	11	41	15	47	42
**Number of bp at location 2-7**	95	9	53	11	10	28	6	31	30
Duplex minimum free energy	29	13	6	9	58	14	15	90	29
Proportion of G in mRNA at the site region	26	21	6	10	21	25	42	64	27
Proportion of GG in mRNA at the site region	67	6	5	11	4	23	33	45	24
**bp in the 4th nt of the seed**	19	29	11	9	6	12	1	21	13
**bp in the 5th nt of the seed**	27	8	8	13	3	12	12	20	13
Number of bulges outside the seed	7	17	3	22	18	7	4	14	12
**Number of GC bp within the seed**	15	6	13	16	7	10	4	20	11
**bp in the 2nd nt of the seed**	18	12	20	6	6	10	6	10	11
Accessibility (nt=21, len=10)	19	5	4	6	14	6	5	11	9
minimum free energy of the target site + 50nt flanking regions	18	3	3	6	5	8	15	10	8
Number of GC bp outside the seed	8	8	6	9	16	6	3	8	8
**Number of mismatches inside the seed**	10	1	8	17	0	10	1	16	8

The table shows 16 features representing the union of the top 6 features of each dataset, along with their gain values which were computed by XGBoost. The features are ordered by their mean gain across all datasets. This is an unscaled version of Table 6.

Table S6. Cross-dataset classification results (complementary information to Figure 7)

	ca1	ce1	ce2	h1	h2	h3	m1	m2
ca1	0.937 (0.002)	0.779 (0.005)	0.733 (0.01)	0.699 (0.003)	0.902 (0.002)	0.839 (0.002)	0.885 (0.006)	0.823 (0.002)
ce1	0.61 (0.005)	0.889 (0.014)	0.809 (0.011)	0.561 (0.006)	0.732 (0.009)	0.575 (0.005)	0.598 (0.008)	0.626 (0.006)
ce2	0.688 (0.008)	0.806 (0.007)	0.891 (0.016)	0.654 (0.008)	0.647 (0.008)	0.667 (0.008)	0.643 (0.01)	0.657 (0.006)
h1	0.794 (0.005)	0.714 (0.007)	0.686 (0.011)	0.824 (0.007)	0.864 (0.006)	0.792 (0.004)	0.815 (0.008)	0.786 (0.004)
h2	0.725 (0.004)	0.712 (0.005)	0.628 (0.007)	0.582 (0.004)	0.904 (0.007)	0.727 (0.004)	0.78 (0.008)	0.753 (0.005)
h3	0.832 (0.004)	0.691 (0.005)	0.654 (0.009)	0.693 (0.006)	0.898 (0.003)	0.835 (0.007)	0.872 (0.007)	0.828 (0.003)
m1	0.782 (0.007)	0.638 (0.01)	0.591 (0.011)	0.623 (0.006)	0.853 (0.006)	0.771 (0.005)	0.847 (0.015)	0.778 (0.005)
m2	0.856 (0.002)	0.705 (0.004)	0.663 (0.005)	0.702 (0.002)	0.908 (0.003)	0.83 (0.003)	0.891 (0.004)	0.9 (0.004)

Each cell *(i,j)* represents the mean accuracy and standard deviation of the 20 classifiers that were trained on dataset *i* and tested on dataset *j ACC(i, j)*.

Table S7. Intra-dataset classification accuracy with 16 features only

Dataset	XGBoost	RF	KNN	SGD	SVM	LR
ca1	0.889 (0.004)	0.889 (0.003)	0.835 (0.003)	0.759 (0.013)	0.842 (0.003)	0.771 (0.005)
ce1	0.851 (0.015)	0.85 (0.014)	0.782 (0.015)	0.778 (0.019)	0.829 (0.015)	0.794 (0.017)
ce2	0.87 (0.017)	0.868 (0.017)	0.809 (0.014)	0.817 (0.032)	0.859 (0.016)	0.837 (0.015)
h1	0.796 (0.007)	0.792 (0.006)	0.748 (0.008)	0.733 (0.011)	0.784 (0.008)	0.743 (0.008)
h2	0.892 (0.012)	0.885 (0.008)	0.852 (0.009)	0.861 (0.021)	0.884 (0.009)	0.881 (0.008)
h3	0.809 (0.009)	0.805 (0.007)	0.761 (0.011)	0.711 (0.047)	0.804 (0.004)	0.754 (0.007)
m1	0.832 (0.013)	0.833 (0.015)	0.746 (0.018)	0.71 (0.071)	0.808 (0.014)	0.762 (0.016)
m2	0.855 (0.004)	0.848 (0.004)	0.808 (0.003)	0.785 (0.015)	0.845 (0.004)	0.794 (0.004)

Intra-dataset classification accuracy performed with different machine learning methods that incorporate only 16 features identified in section *Top important features of each dataset*. The cells contain the means and standard deviations (in brackets) of the accuracy results acquired from 20 models that were trained and evaluated on different training-testing dataset splits.

$$ACC = \frac{TP + TN}{TP + FP + FN + TN}$$ **Equation S1.**

$$TPR = \frac{TP}{TP + FN}$$ **Equation S2.**

$$TNR = \frac{TN}{TN + FP}$$ **Equation S3.**

$$MCC = \frac{TP * TN - FP * FN}{\sqrt{(TP + FP)(TP + FN)(TN + FP)(TN + FN)}}$$ **Equation S4.**

$$F1score = \frac{TP}{TP + \frac{1}{2}(FP + FN)}$$ **Equation S5.**

REFERENCES

1. V. Agarwal, G. W. Bell, J.-W. Nam, and D. P. Bartel. Predicting effective microrna target sites in mammalian mrnas. *elife*, 4:e05005, 2015.
2. C.-H. Chou, N.-W. Chang, S. Shrestha, S.-D. Hsu, Y.-L. Lin, W.-H. Lee, C.-D. Yang, H.-C. Hong, T.-Y. Wei, S.-J. Tu, et al. mirtarbase 2016: updates to the experimentally validated mirna-target interactions database. *Nucleic acids research*, 44(D1):D239–D247, 2016.
3. J. Ding, X. Li, and H. Hu. Tarpmir: a new approach for microrna target site prediction. *Bioinformatics*, 32(18):2768–2775, 2016.
4. S. Grosswendt, A. Filipchyk, M. Manzano, F. Klironomos, M. Schilling, M. Herzog, E. Gottwein, and N. Rajewsky. Unambiguous identification of mirna: target site interactions by different types of ligation reactions. *Molecular cell*, 54(6):1042–1054, 2014.
5. A. Helwak, G. Kudla, T. Dudnakova, and D. Tollervey. Mapping the human mirna interactome by clash reveals frequent noncanonical binding. *Cell*, 153(3):654–665, 2013.
6. M. Khorshid, J. Hausser, M. Zavolan, and E. Van Nimwegen. A biophysical mirna-mrna interaction model infers canonical and noncanonical targets. *Nature methods*, 10(3):253, 2013.
7. W. Liu and X. Wang. Prediction of functional microrna targets by integrative modeling of microrna binding and target expression data. *Genome biology*, 20(1):1–10, 2019.
8. Y. Lu and C. S. Leslie. Learning to predict mirna-mrna interactions from ago clip sequencing and clash data. *PLoS computational biology*, 12(7), 2016.
9. M. J. Moore, T. K. Scheel, J. M. Luna, C. Y. Park, J. J. Fak, E. Nishiuchi, C. M. Rice, and R. B. Darnell. mirna–target chimeras reveal mirna 3-end pairing as a major determinant of argonaute target specificity. *Nature communications*, 6:8864, 2015.
10. A. Paker and H. Oğul. mirlstm: A deep sequential approach to microrna target binding site prediction. In *International Conference on Database and Expert Systems Applications*, pages 38–44. Springer, 2019.
11. A. Pla, X. Zhong, and S. Rayner. miraw: A deep learning-based approach to predict microrna targets by analyzing whole microrna transcripts. *PLoS computational biology*, 14(7):e1006185, 2018.
12. I. S. Vlachos, M. D. Paraskevopoulou, D. Karagkouni, G. Georgakilas, T. Vergoulis, I. Kanellos, I.-L. Anastasopoulos, S. Maniou, K. Karathanou, D. Kalfakakou, et al. Diana-tarbase v7. 0: indexing more than half a million experimentally supported mirna: mrna interactions. *Nucleic acids research*, 43(D1):D153–D159, 2015.
13. X. Wang. Improving microrna target prediction by modeling with unambiguously identified microrna-target pairs from clip-ligation studies. *Bioinformatics*, 32(9):1316–1322, 2016.
14. M. Wen, P. Cong, Z. Zhang, H. Lu, and T. Li. Deepmirtar: a deep-learning approach for predicting human mirna targets. *Bioinformatics*, 34(22):3781–3787, 2018.
15. F. Xiao, Z. Zuo, G. Cai, S. Kang, X. Gao, and T. Li. mirecords: an integrated resource for microrna–target interactions. *Nucleic acids research*, 37(suppl_1):D105–D110, 2009.
